# Chemical standardization of kava (*Piper methysticum*) extract using UHPLC-MS/MS and the method of standard addition

**DOI:** 10.1080/13880209.2026.2727761

**Published:** 2026-09-11

**Authors:** Ruth N. Muchiri, Natalie R. Thun, Richard B. van Breemen

**Affiliations:** Linus Pauling Institute, Department of Pharmaceutical Sciences, College of Pharmacy, Oregon State University, Corvallis, Oregon, USA

**Keywords:** Kava, *Piper methysticum*, method of standard addition, matrix effects, electrospray UHPLC-MS/MS, quantitative analysis

## Abstract

**Background:**

Kava (*Piper methysticum* G.Forst.) is used traditionally in the South Pacific for ceremonial and medicinal purposes and as a dietary supplement for its anxiolytic effects. The pharmacological activity of kava is attributed to kavalactones and flavokavains, including desmethoxyyangonin, 7,8-dihydrokavain, kavain, methysticin, 7,8-dihydromethysticin, and yangonin. The safe and effective use of kava dietary supplements depends on botanical authentication and standardization to these active constituents.

**Objective:**

To support safety studies being carried out by the Botanical Safety Consortium, the chemical profile of a botanically authenticated kava extract was obtained using ultrahigh-pressure liquid chromatography-high resolution tandem mass spectrometry (UHPLC-HRMS/MS), and kavalactones and flavokavains were identified by comparison with standards.

**Materials and Methods:**

The kava extract was chemically standardized using two analytical methods based on UHPLC-MS/MS with selected reaction monitoring on a triple quadrupole mass spectrometer. One method used external calibration with standards dissolved in neat solvent due to the absence of blank kava matrix, while the second used the method of standard addition to evaluate kava extract matrix effects that might affect the accuracy of the external calibration method.

**Results:**

Matrix-induced ion suppression was observed for desmethoxyyangonin (23.2%) while signal enhancement was observed for 7,8-dihydrokavain (30.1%) and flavokavain A (43.2%).

**Discussion and Conclusions:**

The method of standard addition is ideal for correcting for botanical matrix effects that can interfere with quantitative assays based on electrospray (U)HPLC-MS/MS. Chemical authentication and standardization are essential for quality control of kava dietary supplements and to support research on their safety and efficacy.

## Introduction

Water extracts of the ground roots and rhizomes of kava (*Piper methysticum* G.Forst.) also called kava kava, are traditional beverages in the Pacific islands that have psychoactive properties, particularly anxiolytic and relaxation effects (Pittler and Ernst 2000; Bilia et al. 2002; Savage et al. 2015; Kuchta et al. 2021; Devi and Pasinszki 2025). As a dietary supplement, kava roots and rhizomes are typically extracted using an organic solvent and has become a top selling product in the United States with sales increasing 22.5% during 2023 and 4.8% in 2024 (Smith et al. 2025). The primary bioactive compounds are kavalactones and flavokavains, which comprise 30% to 90% of kava extracts by weight in dietary supplements (Côté et al. 2004; Pasinszki et al. 2025). With the growing popularity of kava-based supplements for therapeutic and recreational purposes, there is an increasing need for chemical standardization to ensure product consistency, safety, and efficacy (Sarris et al. 2011; Kuchta et al. 2015; Thomsen and Schmidt 2021). Accurate identification and chemical standardization of kavalactones in kava dietary supplements are critical for quality control, regulatory compliance, and the accurate assessment of their biological activities (van Breemen et al. 2007; Kuchta et al. 2015).

**Figure 1.**
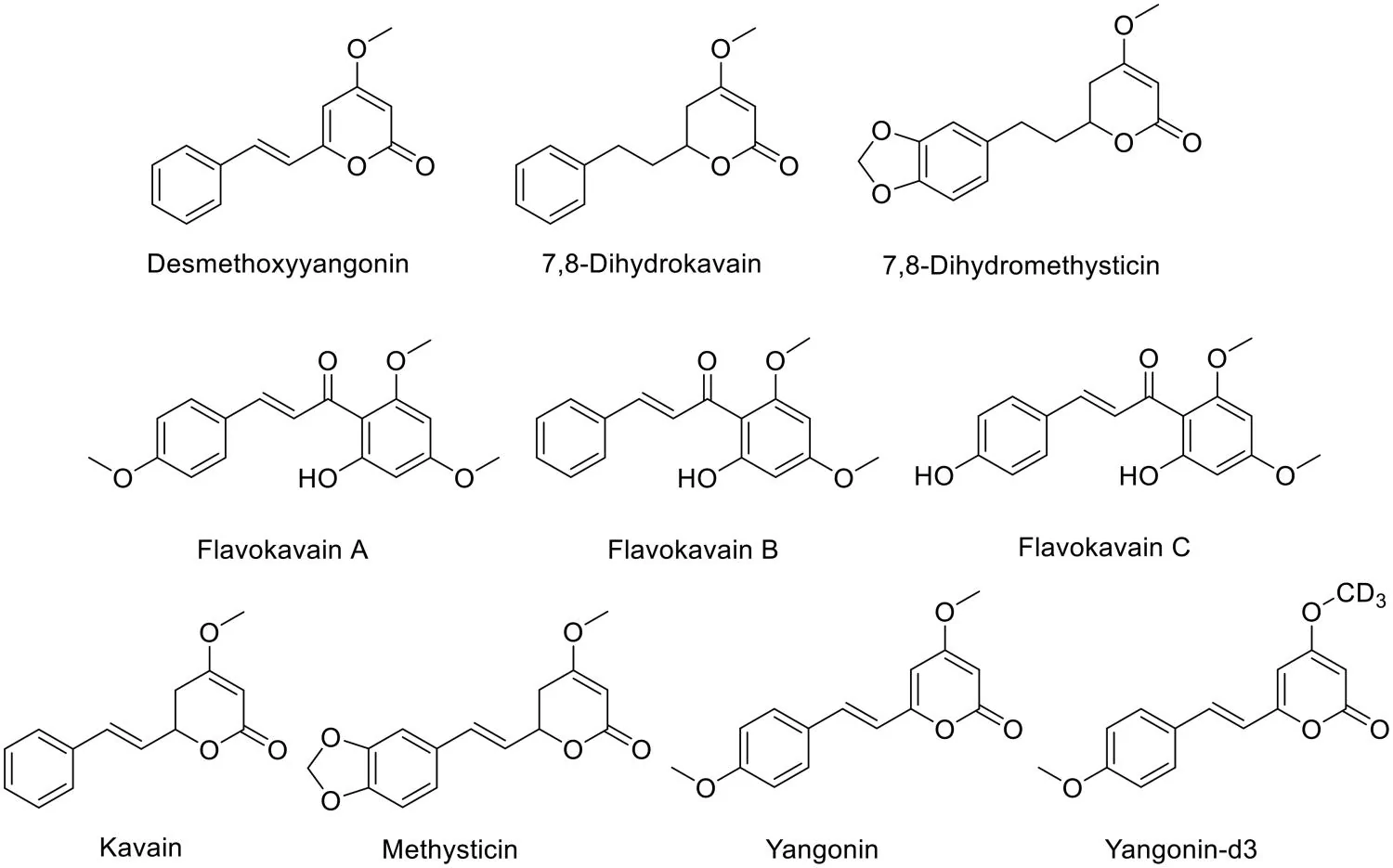
Chemical structures of kavalactones, flavokavains and deuterated internal standard used for standard curves.

Methods for the chemical standardization of kava dietary supplements have included high-performance liquid chromatography (HPLC) with UV absorbance detection (Liu et al. 2018; Ferreira et al. 2021; Aijaz et al. 2024), ultrahigh pressure liquid chromatography (UHPLC) with UV absorbance detection (Tang and Fields 2019), supercritical fluid chromatography with UV absorbance detection (Murauer and Ganzera 2017), HPLC-mass spectrometry (LC-MS) (De Jager et al. 2004). HPLC-tandem mass spectrometry (MS/MS) (Wang et al., 2018), and UHPLC-tandem mass spectrometry (MS/MS) (Mamallapalli et al. 2022). These methods enable the identification and quantification of individual kavalactones and flavokavains such as desmethoxyyangonin, 7,8-dihydrokavain, kavain, methysticin, 7,8-dihydromethysticin, yangonin, flavokavain A, flavokavain B, and flavokavain C (Figure 1), which are critical markers for both dietary supplement quality and therapeutic potential. Chromatographic separation times for kava extracts have ranged from 5 min to 18 min, with UHPLC providing the fastest separation. However, none of these assays were validated using the method of standard addition, which can determine whether matrix effects might affect the accuracy of the analytical measurements (Hewavitharana et al. 2018).

During the method of standard addition, calibration standards are spiked into aliquots of the sample containing unknown concentrations of the analytes, and the mixture is processed and measured (Muchiri and van Breemen 2024). The method of standard addition corrects for any matrix effects and sample loss during preparation and is ideal when no blank matrix is available. However, this approach requires more sample and more analyses per sample than the external calibration curve method, which can limit the method of standard addition to the analysis of small numbers of samples.

When standardizing botanical dietary supplements, matrix effects can be problematic as no blank matrix exists for use in the preparation of standard curves (Muchiri and van Breemen 2024). During HPLC-UV or supercritical fluid chromatography-UV, coeluting compounds can contribute to analyte signals, and during electrospray HPLC-MS and UHPLC-MS/MS, coeluting compounds can suppress or enhance analyte signals. In this study, two UHPLC-MS/MS assays were compared. Both used UHPLC instead of HPLC for faster separation, but while one assay used external calibration with standards dissolved in neat solvent, the other used the method of standard addition.

## Methods

### Materials and reagents

Desmethoxyyangonin (CDX-00004236, 99.7% purity), 7,8-dihydrokavain (CDX-00004476, 99.7% purity), flavokavain B (CDX-00006058, ≥99.0% purity), kavain (CDX-00011300, ≥99.9% purity), methysticin (CDX-00013860, ≥99.7% purity), and yangonin (CDX-00025010, ≥99.1% purity) were purchased from ChromaDex (Los Angeles, CA, USA). Flavokavain A, (TRC-H214235, 98.0% purity), and 7,8-dihydromethysticin (TRC-B203530, 98.0% purity) were purchased from LGC Standards (Toronto, Canada). Yangonin-*d_3_* (sc-475707, purity 99.7%) was purchased from Santa Cruz Biotechnology (Santa Cruz, CA, USA), and daidzein-*d_4_* (18249, purity <99%) was purchased from Cayman Chemical (Ann Arbor, MI, USA). An ethanolic extract of botanically authenticated kava roots and rhizomes was supplied by the Botanical Safety Consortium (National Toxicology Program \(NTP\), 2023). HPLC-MS grade acetonitrile, methanol, and formic acid were purchased from Thermo Fisher Scientific (Waltham, MA, USA). Ultrapure water was prepared using a MilliporeSigma Milli-Q water purification system. All other reagents and solvents were reagent grade or better and were purchased from VWR (Visalia, CA, USA).

### Preparation of calibration standards and internal standards

The stock solutions of kavalactone and flavokavain standards (1 mg/mL each) were prepared in methanol, aliquoted, and stored at −20 °C until use. The working solutions for the calibration standards were prepared by diluting the stock solution in methanol/water (1:1, v/v). The stock solution of internal standard yangonin-*d_3_* (2.5 mg/mL) was prepared in water/dimethylsulfoxide (1:1, v/v) and then diluted in methanol/water (1:1, v/v) to obtain a working solution (77.8 ng/mL) for addition during sample pretreatment.

### Standard addition method and external calibration curve

The kava extract was weighed and dissolved in methanol to prepare a 1 mg/mL stock solution, which was diluted to 0.5 µg/mL using 50% aqueous methanol. To construct a standard addition calibration curve, known amounts of kavalactone and flavokavain standards were spiked into aliquots of the diluted kava extract. For comparison, an external calibration curve was created by spiking the standards into neat solvent (50% aqueous methanol) at varying concentrations. The concentration ranges for the standards were as follows: 0.4–256 ng/mL for desmethoxyyangonin, 7,8-dihydrokavain, flavokavain A, kavain, methysticin, 7,8-dihydromethysticin, and yangonin, and 0.2–128 ng/mL for flavokavain B. The concentration of internal standard yangonin-*d3* in all samples was 50 ng/mL.

### Stability assays of kavalactone and flavokavains

Quality control standards at the lower limit of quantitation (LLOQ), low, medium and high concentrations were prepared by diluting the stock solutions in methanol/water (1:1, v/v). The stability of each analyte was evaluated at the auto-sampler temperature of 15 °C for 24 h and for 2 freeze-thaw cycles with a 14-day interval.

### High-resolution UHPLC-MS and UHPLC-MS/MS

High-resolution mass spectra and data-dependent tandem mass spectra of kavalactones and flavokavains in the kava extract were obtained using a Shimadzu (Kyoto, Japan) 9030 Q-ToF tandem mass spectrometer interfaced with a Shimadzu Nexera UHPLC system. Chromatographic separation of the kavalactones and flavokavains was carried out using a Waters (Milford, MA, USA) Cortecs UPLC C_18_ column (1.7 μm, 130 Å, 2.1 × 150 mm). The mobile phase consisted of a gradient from water (A) to methanol (B) both containing 0.1% formic acid as follows: 0-10 min 5%-40% B, 10-15 min 40% B, 15-17 min 40-45% B, 17-19 min 45-50% B, 19-21 min 50% B, 21-25 min 50-90% B. The column was washed with 95% B for 2 min and reconditioned in 5% methanol between injections. The flow rate was 0.3 mL/min, the injection volume was 10 µL, and the column and autosampler temperatures were 45 °C and 15 °C respectively. Daidzein-*d_4_* was added to the extract immediately before analysis as an injection standard.

The electrospray ionization interface temperature was 300 °C, and the voltage was 4.0 kV for positive ion mode. The heat block and desolvation line temperatures were 400 °C and 250 °C, respectively. Nitrogen was used as a drying gas at a flow rate of 10 L/min, for nebulization at 3 L/min, and as a heating gas at 10 L/min. High resolution mass spectra and product ion tandem mass spectra were acquired every 100 ms over the mass range *m*/*z* 75 – 2000. Product ion tandem mass spectra were obtained using a collision energy of 35 V with an energy spread of 17 V.

### Quantitative UHPLC-MS/MS

For quantitative analysis, the UHPLC system was interfaced with a Shimadzu LCMS-8060 triple quadrupole mass spectrometer equipped with positive ion electrospray ionization. Chromatographic separation was carried out using a Waters Acquity BEH UPLC C_18_ column (1.7 μm, 2.1 × 100 mm). The mobile phase was the same as described above but used a shorter gradient as follows: 0-4 min 60-80% B, 4-5 min 80-98% B, 5-6 min 98% B. The flow rate was 0.3 mL/min, the injection volume was 3 µL, and the column and autosampler temperatures were 40 °C and 15 °C respectively. The total UHPLC analysis time per sample for the separation of kavalactones was 8 min. Nitrogen was used as drying gas at a flow rate of 5 L/min and for nebulization at 3 L/min. The interface and desolvation line temperatures were 400 °C and 300 °C, respectively. The kavalactones and flavokavains were measured using collision-induced dissociation with selected reaction monitoring (SRM). The SRM dwell time for each transition was 19 msec, and the collision gas pressure was 230 kPa. Data acquisition, integration, and linear standard curve fitting were carried out using Shimadzu Lab Solutions version 5.7. The concentration of kavalactones and flavokavains in the kava extract was calculated using Microsoft Excel (Seattle, WA, USA).

## Results and discussion

### Chemical authentication of kava extract

**Figure 2.**
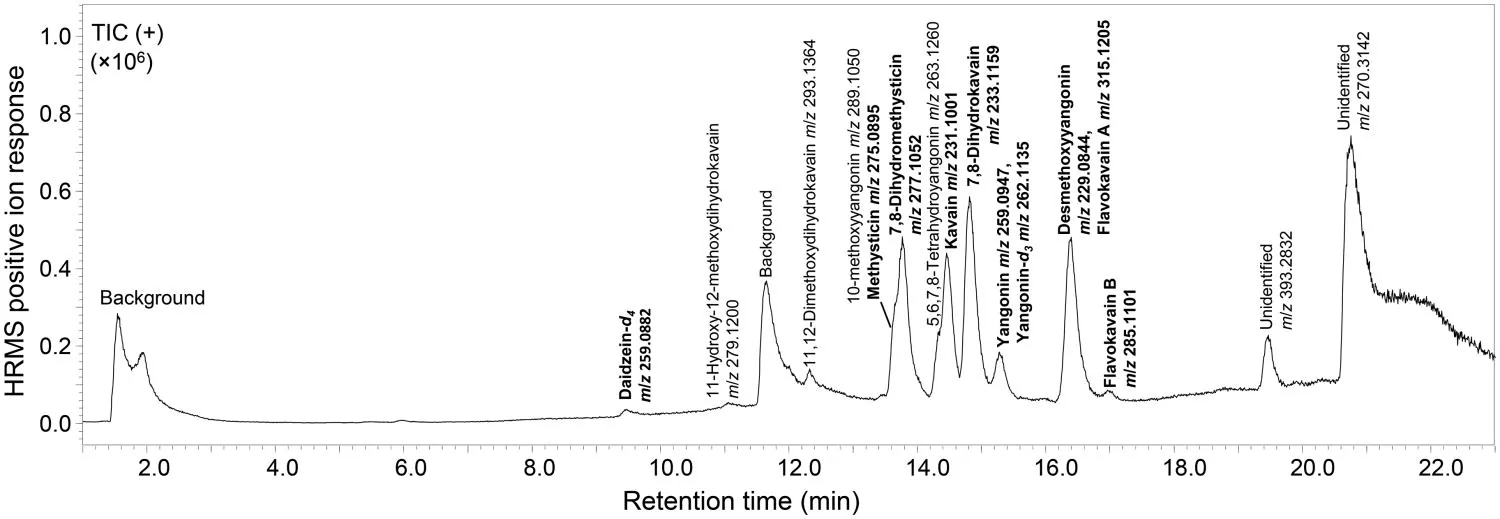
High-resolution positive ion electrospray UHPLC-MS total ion chromatogram of the chemically standardized kava extract confirming identification of all the major constituents.

To provide chemical authentication of the kava extract used in this study (National Toxicology Program \(NTP\) 2023; Patel et al. 2023), the extract was profiled using high-resolution UHPLC-MS and MS/MS with positive ion electrospray to verify the presence of characteristic kavalactones and flavokavains (Figure 2). Using this untargeted approach, all intense UHPLC-MS peaks were characterized to confirm the identity of the botanical while helping to detect possible adulteration, contamination, or inadvertent substitution with non-kava materials. For example, consumption of a kava product containing undisclosed kratom was reported recently in a clinical case study to have caused opioid-like withdrawal symptoms (Malhotra et al. 2026). Based on accurate mass measurement, tandem mass spectrometry, UHPLC retention times, and comparison with standards, methysticin (retention time 13.67 min), 7,8-dihydromethysticin (13.79 min), kavain (14.48 min), 7,8-dihydrokavain (14.86 min), yangonin (15.33 min), desmethoxyyangonin (16.35 min), flavokavain A (16.61 min), and flavokavain B (17.02 min) were identified in the kava extract (Figure 2, Supplemental Table S1, and Supplemental Figures S1–S18). No peak was detected corresponding to flavokavain C in this kava extract. Although no standards were available for comparison, the analytes eluting at 10.97 min and 14.32 min were assigned to the kava constituents 11-hydroxy-12-methoxydihydrokavain and 5,6,7,8-tetrahydroyangonin, respectively, based on accurate mass measurements (Table S1) and comparison of product ion tandem mass spectra (Figure S3 and S9) with those in the literature (He et al. 1997; Tang and Fields 2019). The analytes eluting at 12.38 min and 13.59 min corresponded to the kava constituents 11,12-dimethoxydihydrokavain and 10-methoxyyangonin based on accurate mass measurements (Table S1) and product ion tandem mass spectra (Figures S5 and S6), which showed class-characteristic fragment ions consistent with other kavalactones and flavokavains. UHPLC-MS profiling confirmed the identity of the botanical in this extract to be kava, and no evidence of adulteration or contamination with non-kava botanicals or other materials was found.

**Figure 3.**
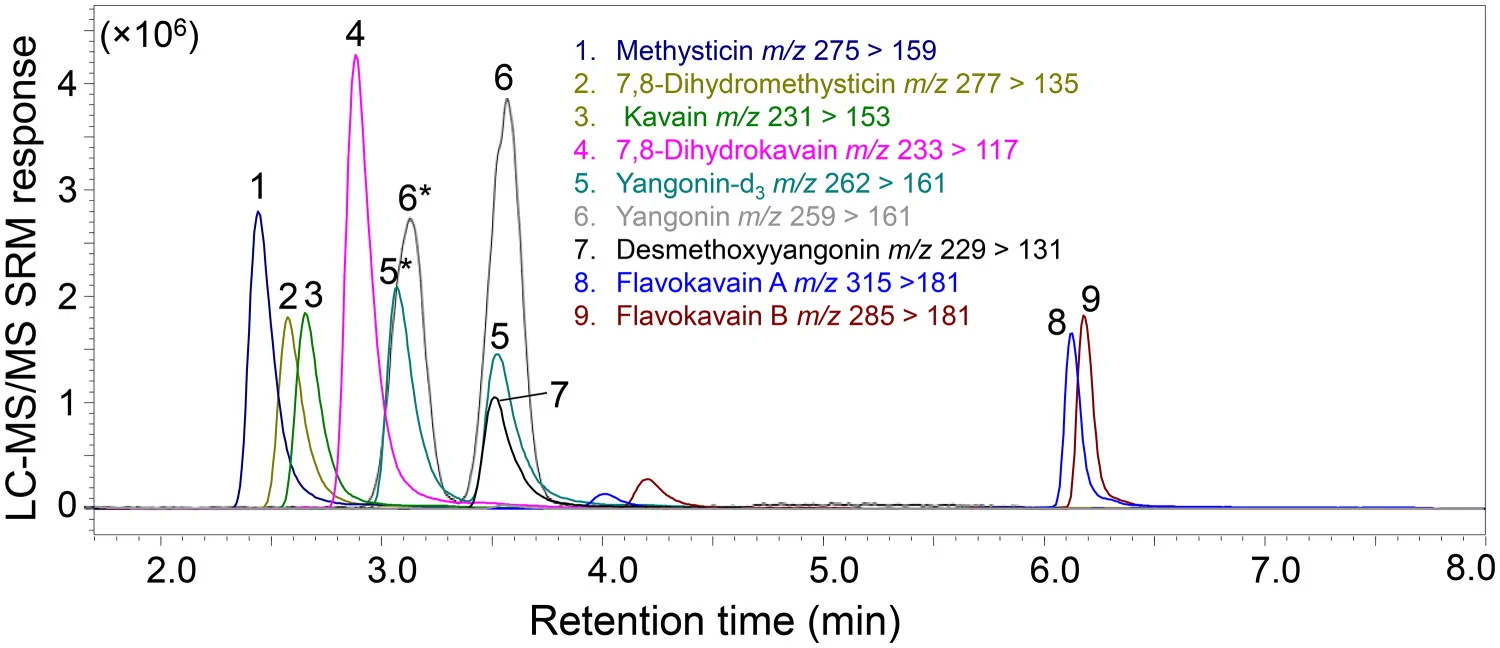
Positive ion electrospray UHPLC-MS/MS selected reaction monitoring (SRM) chromatograms of kavalactones and flavokavains in an ethanolic extract of kava (0.5 µg/mL). Yangonin-*d_3_* was used as internal standard. *cis-yangonin-*d_3_* and *cis-yangonin.

### Stabilities of kavalactone and flavokavains

To ensure accuracy, the stabilities of the kavalactones and flavokavains were evaluated under the assay conditions (Table 1). Kavalactones are stable under neutral and mildly acidic conditions but may degrade at elevated temperatures or during prolonged light exposure, but flavokavains are less stable under similar conditions, particularly in the presence of oxygen and light (Tang and Fields 2019). Stabilities of these kava compounds in the autosampler were determined by analyzing freshly prepared standards and reinjecting the processed samples after 24 h in the autosampler at 15 °C. Freeze-thaw stabilities were determined at each quality control concentration following storage at −20 °C for 36 h and thawing at room temperature. After thawing, aliquots were removed and kava compounds were measured by using UHPLC-MS/MS, and the remaining samples were refrozen and stored at −20 °C for 2 weeks. The samples were thawed again at room temperature and reanalyzed.

**Table 1.** Stabilities of kavalactones and flavokavains during storage and handling, measured using UHPLC-MS/MS.

Analyte (*n* ≥ 3)	QC level	Theoretical (ng/mL)	Autosampler 24 h (% of theoretical)	Freeze-Thaw (% of theoretical)	
				1st	2nd
Desmethoxyyangonin	LLOQ	0.4	106 ± 3	106 ± 7	99.6 ± 6.3
	Low	5	99.5 ± 10.2	103 ± 3	102 ± 3
	Mid	50	99.0 ± 3.4	103 ± 2	95.6 ± 8.2
	High	250	103 ± 3	105 ± 2	99.9 ± 10.0
7,8-Dihydrokavain	LLOQ	0.4	104 ± 4	100 ± 7	89.1 ± 6.0
	Low	5	101 ± 7	101 ± 1	105 ± 4
	Mid	50	104 ± 4	104 ± 2	93.6 ± 6.9
	High	250	104 ± 3	104 ± 2	84.9 ± 6.1
Flavokavain A	LLOQ	0.4	92.3 ± 8.5	101 ± 10	92.2 ± 6.0
	Low	5	92.5 ± 9.1	105 ± 4	110 ± 2
	Mid	50	95.2 ± 8.6	109 ± 3	97.1 ± 4.9
	High	250	100 ± 3	104 ± 1	114 ± 11
Flavokavain B	LLOQ	0.2	90.2 ± 7.8	108 ± 9	106 ± 12
	Low	2	89.1 ± 7.0	116 ± 3	115 ± 3
	Mid	25	88.3 ± 9.8	123 ± 4	111 ± 6
	High	125	94.3 ± 4.3	111 ± 1	102 ± 10
Kavain	LLOQ	0.4	107 ± 10	102 ± 9	96.4 ± 6.1
	Low	5	105 ± 10	107 ± 3	109 ± 2
	Mid	50	101 ± 3	104 ± 3	96.4 ± 6.4
	High	250	103 ± 4	104 ± 1	96.0 ± 7.1
Methysticin	LLOQ	0.4	98.2 ± 9.0	103 ± 4	104 ± 8
	Low	5	100 ± 6	102 ± 2	110 ± 2
	Mid	50	104 ± 4	108 ± 2	98.6 ± 7.2
	High	250	103 ± 3	107 ± 1	95.0 ± 6.8
7,8-Dihydromethysticin	LLOQ	0.4	98.6 ± 8.0	114 ± 3	101 ± 14
	Low	5	98.0 ± 6.7	108 ± 2	106 ± 6
	Mid	50	100 ± 6	110 ± 2	96.9 ± 8.0
	High	250	103 ± 6	110 ± 2	93.2 ± 6.8
Yangonin	LLOQ	8	98.2 ± 13.4	98.1 ± 8.9	84.3 ± 6.5
	Low	40	94.7 ± 9.2	103 ± 10	92.2 ± 7.3
	Mid	400	96.8 ± 8.0	104 ± 3	93.5 ± 6.6
	High	2000	101 ± 5	107 ± 2	91.7 ± 7.5

The compounds were determined to be stable if the differences between freshly prepared samples and those tested after treatment were less than 15%. Based on these criteria, all the kavalactone and flavokavain analytes were stable under the testing and storage conditions (Table 1). During the actual analysis, the kava extracts were analyzed within 24 h without freeze-thawing.

High-resolution positive ion electrospray mass spectrometry was used to verify that the isotopic purity of the yangonin-*d_3_* internal standard was >99% and free from unlabeled yangonin. This is important because the addition of unlabeled yangonin along with the labeled yangonin internal standard would result in an inaccurately high value for yangonin when using the method of standard addition. Yangonin-*d_3_* and the unlabeled yangonin standard each produced a single chromatographic peak for their trans isomers during UHPLC-MS/MS with SRM, but after spiking into the kava extract, two peaks were detected for each, corresponding to the cis- and trans isomers (Figure 3). Although cis/trans isomerization of yangonin has been reported to occur upon exposure to heat or light (Bobeldijk et al. 2005), it was an unexpected matrix effect that the extract itself catalyzed rapid isomerization of this compound. Isomerization of other kava compounds was not observed. Because *cis*-yangonin was only observed when using the method of standard addition, the sum of both *cis*- and *trans*-yangonin was used to compare the total yangonin determined using the two analytical methods.

### Chemical standardization of kava extract

A quantitative assay based on positive-ion electrospray UHPLC-MS/MS with SRM was developed for the measurement of 8 kavalactones and flavokavains in an ethanolic extract of kava. The two most abundant product ions of each protonated analyte were identified using pure standards at 1 µg/mL, and the collision energies that produced the most abundant of these product ions were determined (Table 2). The quantifier SRM transition was selected as the most abundant product ion of each protonated molecule, while the second most abundant product ion formed from the protonated molecule served as the qualifier SRM transition.

**Table 2.** UHPLC-MS/MS positive ion electrospray retention times (RT), MS/MS selected reaction monitoring (SRM) transitions, collision energies (CE), lower limits of quantitation (LLOQ), coefficients of determination (R^2^), and concentrations of standards (ng/mL) prepared either in neat solvent or using the method of standard addition.

Analyte	RT (min)	SRM transitions *m*/*z*	CE (V)	LLOQ	Spiked concentrations (ng/mL)									R^2^ Neat solvent	R^2^ Standard addition
Desmethoxyyangonin	3.51	229 → 131^a^ 229 → 141	−20 −23	0.4	0.4	2	4	8	16	32	64	128	256	0.9998	0.9990
7,8-Dihydrokavain	2.89	233 → 117 233 → 91	−21 −34	0.4	0.4	2	4	8	16	32	64	128	256	0.9933	0.9977
Flavokavain A	6.14	315 → 181 315 → 161	−22 −20	0.4	0.4	2	4	8	16	32	64	128	256	0.9936	0.9979
Flavokavain B	6.20	285 → 181 285 → 138	−21 −38	0.2	0.2	0.4	2	4	8	16	32	64	128	0.9937	0.9978
Kavain	2.66	231 → 153 231 → 115	−21 −14	0.4	0.4	2	4	8	16	32	64	128	256	0.9914	0.9995
Methysticin	2.44	275 → 159 275 → 103	−15 −36	0.4	0.4	2	4	8	16	32	64	128	256	0.9940	0.9985
7,8-Dihydromethysticin	2.57	277 → 135 277 → 161	−22 −13	0.4	0.4	2	4	8	16	32	64	128	256	0.9880	0.9985
Yangonin	3.57	259 → 161 259 → 133	−21 −31	0.4	0.4	2	4	8	16	32	64	128	256	0.9996	0.9998
Yangonin-*d*_3_ Internal standard	3.54	262 → 161 262 → 133	−21 −31	–	–	–	–	–	–	–	–	–	–	–	–

The UHPLC separation was optimized for separation of the kava analyte mixture within 8 min (Figure 3). As for most dietary supplements, no blank matrix for kava was available. Therefore, standards for the external calibration curve were initially prepared in 50% aqueous methanol. Using these standard solutions, calibration curves of all 8 kavalactones and flavokavains were linear over 3 orders of magnitude with R^2^ ≥ 0.99 (Table 2). The 8 kava compounds were then measured in the extract (Figure 3, Table 4), and kavain (130.2 ± 5.5 mg/g) was the most abundant analyte, while flavokavain A (8.346 ± 1.174 mg/g) was the least abundant. The sum of all 8 major kavalactones and flavokavains in the kava root and rhizome extract was 556.9 mg/g (55.69%), which is within the expected range of 30% to 90% by weight (Côté et al. 2004).

Interday and intraday precision and accuracy were determined at each quality control concentration (Table 3), and the precision was within 15% for all compounds at all concentrations. The method was accurate except that at the highest concentrations tested, the electrospray signal started to saturate and the curve deviated from linearity.

**Table 3.** Precision and accuracy for measurement of kava lactones and flavokavains using the external calibration method.

		Precision (*n* = 5) (%Coefficient of variation)		Accuracy (*n* = 3) (RE%)	
Analyte	Theoretical (ng/mL)	Intraday	Interday	Intraday	Interday
Desmethoxyyangonin	0.5	3.72	8.38	94.6	101.8
	5	6.44	7.69	106.0	116.2
	50	3.12	5.00	113.2	120.2
	250	5.34	6.77	117.0	121.9
7,8-Dihydrokavain	0.5	3.88	9.35	94.0	90.7
	5	3.81	6.10	114.4	121.5
	50	1.13	4.11	106.0	111.5
	250	4.08	5.46	126.0	129.5
Flavokavain A	0.5	9.54	15.2	94.0	108.0
	5	7.41	9.58	102.2	97.1
	50	5.27	7.24	107.5	115.0
	250	2.09	4.08	75.4	75.3
Flavokavain B	0.2	12.0	15.9	90.0	88.3
	2	5.60	10.8	106.0	97.0
	25	2.83	11.5	103.5	95.6
	150	2.84	6.14	104.0	101.5
Kavain	0.5	10.8	10.2	102.0	98.7
	5	3.87	7.54	105.0	115.0
	50	1.71	5.08	105.8	112.3
	250	2.90	6.63	118.9	124.6
Methysticin	0.5	2.49	11.4	84.0	91.4
	5	5.18	6.81	109.2	116.9
	50	0.912	5.90	102.8	111.0
	250	2.60	6.75	116.1	123.4
7,8-Dihydromethysticin	0.5	6.21	11.9	96.0	101.4
	5	2.45	6.97	115.8	122.9
	50	1.99	6.06	112.9	118.8
	250	1.66	6.09	121.7	126.8
Yangonin	8	13.6	15.5	102.1	99.4
	40	5.09	7.29	107.0	104.8
	400	1.59	6.16	110.3	108.8

Botanical extracts are complex matrices that can cause suppression or enhancement of analyte signals during electrospray mass spectrometry (Annesley 2003; Chambers et al. 2007; Muchiri and van Breemen 2024). Using standards prepared in the biological matrix for external calibration curves can help control for matrix effects, but no blank kava matrix was available. To test the impact of matrix effects on the accuracy of the analytical method developed using neat solvent for the external standards, the method of standard addition was used to measure the same 8 kava compounds with the standard dissolved in the kava root and rhizome extract (Furey et al. 2013).

**Figure 4.**
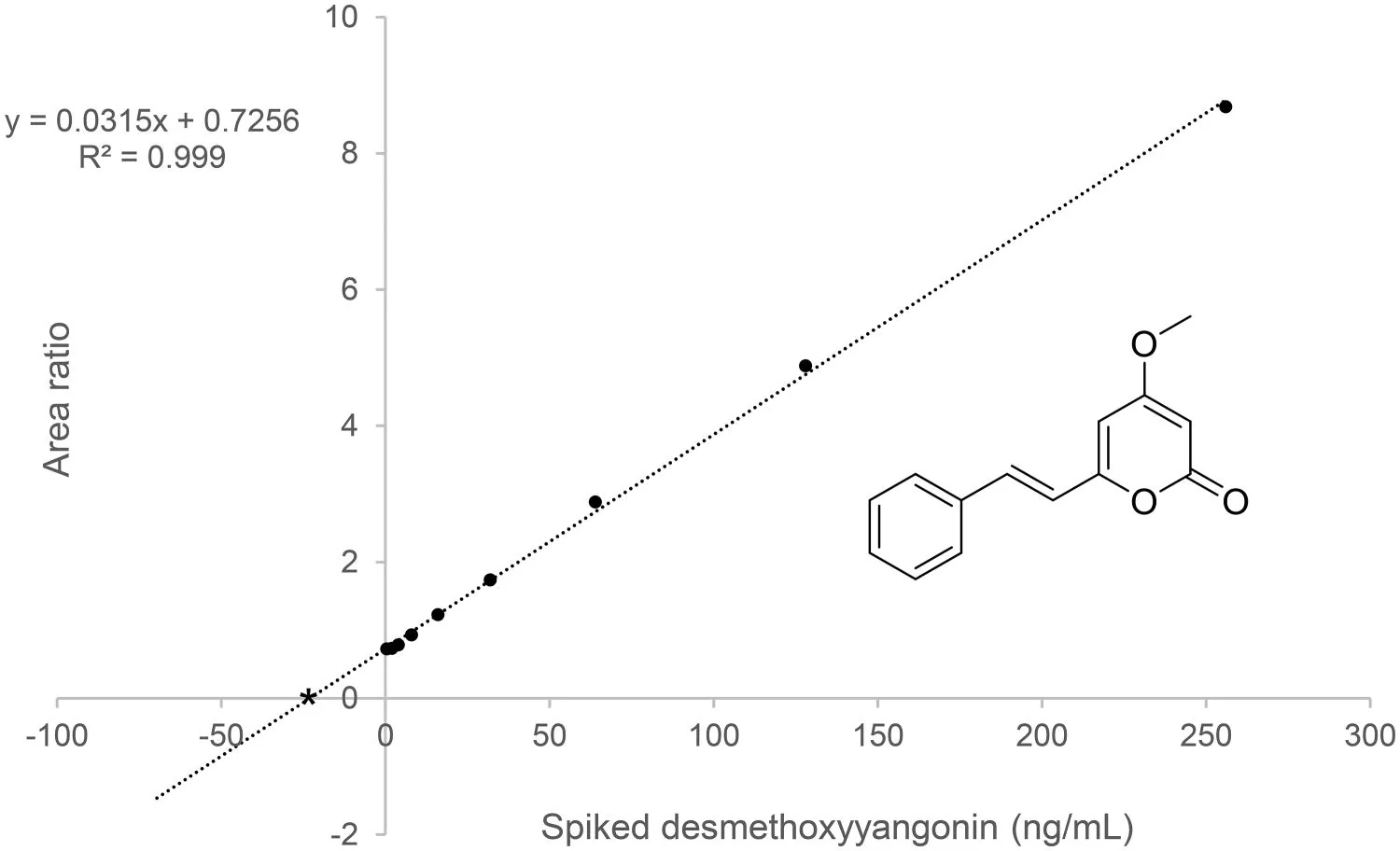
Method of standard addition calibration curve for the UHPLC-MS/MS SRM measurement of desmethoxyyangonin. The concentration of desmethoxyyangonin in the kava extract was derived by extrapolating the linear regression line to the (negative) x-axis, with the absolute value representing the endogenous concentration of the analyte in the kava extract.

For the method of standard addition, aliquots of the kava extract were diluted to a concentration of 0.5 μg/mL so that the UHPLC-MS/MS signals for the endogenous kavalactones and flavokavains produced signal-to-noise ratios of ∼10:1. Standards of the 8 analytes were spiked into the diluted extract at various concentrations (Table 2). Following UHPLC-MS/MS analysis with SRM, the internal standard-normalized peak area for each kavalactone-spiked sample was plotted on the y-axis, while the known concentrations of each spiked standard were plotted on the x-axis. The linear regression line was extrapolated to the (negative) x-axis, with the absolute value representing the endogenous concentration of the respective kavalactone or flavokavain in the kava extract (as an example, see analysis of desmethoxyyangonin in Figure 4). Using the method of standard addition, all 8 analytes produced linear standard curves over 3 orders of magnitude with R^2^ values >0.99 (Table 2).

Both analytical methods produced similar values (within 10%) for flavokavain B, kavain, methysticin, 7,8-dihydromethysticin, and yangonin. However, matrix effects caused significant ion enhancement for 7,8-dihydrokavain (30.1%) and flavokavain A (43.2%), while ion suppression was observed for desmethoxyyangonin (-23.2%) (Table 4) For this kava extract, only the method of standard addition produced accurate results for all 8 of the kavalactones and flavokavains.

**Table 4.** Quantitative analysis of kavalactones and flavokavains in kava extract by UHPLC-MS/MS employing either the method of standard addition or an external calibration curve prepared in neat solvent. The percent differences in analyte levels between the two methods indicate severity of matrix effects.

	Analyte/Kava extract (mg/g)		
Kava compound	Standard addition (*n* = 12; SD)	Neat solvent standard curve (*n* = 3; SD)	% difference
Desmethoxyyangonin	51.19 ± 3.79	39.33 ± 1.94	−23.2
7,8-Dihydrokavain	112.5 ± 4.4	146.3 ± 2.3	30.1
Flavokavain A	8.35 ± 1.17	11.95 ± 0.50	43.2
Flavokavain B	16.15 ± 0.12	15.71 ± 0.10	−2.7
Kavain	130.2 ± 5.5	125.4 ± 2.9	−3.7
Methysticin	54.59 ± 2.58	58.34 ± 0.54	6.9
7,8-Dihydromethysticin	75.60 ± 3.68	78.05 ± 1.59	3.2
Yangonin	108.3 ± 9.49	97.96 ± 3.87	−9.6

Due to the complexity of most botanical extracts and the lack of availability of blank matrices for matrix-matched calibration, matrix effects are likely to occur during quantitative analysis due to co-eluting substances during chromatography (Nasiri et al. 2021). Overlapping compounds can be minimized by using more efficient UHPLC columns instead of HPLC and by including long gradients (Tang and Fields 2019). However, most published quantitative methods for the measurement of kavalactones and kavains in kava products have used HPLC (De Jager et al. 2004; Liu et al. 2018; Wang et al. 2018; Ferreira et al. 2021; Aijaz et al. 2024). During this investigation, UHPLC was used to maximize chromatographic efficiency while maintaining a short gradient so as to complete each measurement within 8 min.

To help correct for matrix effects, sample loss during processing, and changes in instrument response between analyses, stable isotopically labeled analogs of each analyte can be added to samples as surrogate standards (Hewavitharana et al. 2018). Stable isotopically labeled surrogate standards are expensive and were not available for all analytes in this study, so only deuterated yangonin was used as an internal standard. Note that if stable isotopically labeled analogs of an analyte are used for the method of standard addition, they must be isotopically pure. Otherwise, any unlabeled analyte in the internal standard will contribute to the analyte signal and cause a systematic error when using the method of standard addition. Interestingly, isotopic impurities in surrogate standards do not produce such systematic errors in the external standard method because they are added to both samples and calibration standards.

Another approach to minimize matrix effects is to remove the interfering substances prior to analysis (Cortese et al. 2020). These sample clean-up procedures include solid-phase extraction and liquid-liquid extraction. Sample clean-up must be optimized to minimize loss of analyte while removing as much of the interfering substances as possible. Based on the method of standard addition results, we know that additional sample clean-up might improve the accuracy of the measurement of kavalactones and flavokavains using the external calibration method. However, the method of standard addition corrected for this issue without the need for additional method development.

If any kavalactones or flavokavains were more or less stable in the plant matrix than in neat solvent, then this could explain different results between the method of standardization and the external standard method. In the case of different stabilities of kava compounds in matrix versus neat solvent, the method of standard addition should give more accurate results because the standards are added to the matrix and measured simultaneously with the unknowns instead of being prepared in neat solvent and measured separately.

## Conclusions

Matrix effects significantly impacted the accuracy of measurement of several major kavalactones and flavokavains in an extract of kava roots and rhizomes using UHPLC-MS/MS with an external standard curve. These findings highlight the importance of accounting for matrix effects when analyzing kava extracts, particularly through approaches like the method of standard addition. Addressing matrix interferences is essential for ensuring reliable quantification and consistent chemical standardization of kavalactones and flavokavains in kava products.

## Supplementary Material

Supporting Information Kava HR UHPLCMSMS rev.pdf

## Data Availability

Additional data supporting the results presented in this paper are available by request from the corresponding author.
