# Supplementary material for: Chemical standardization of kava (Piper methysticum) extract using UHPLC-MS/MS and the method of standard addition
Source: Pharm Biol. 2026 Sep 11;64(1):1034–44. doi: 10.1080/13880209.2026.2727761 (PMC13573506; doi:10.1080/13880209.2026.2727761)
Supplement: Supplementary file 1 [file IPHB_A_2727761_SM3978.pdf]

Supporting information

Chemical Standardization of Kava (*Piper methysticum*) Extracts Using UHPLC-MS/MS and the Method of Standard Addition

Ruth N. Muchiri, Natalie R. Thun and Richard B. van Breemen*

Linus Pauling Institute, Department of Pharmaceutical Sciences, College of Pharmacy, Oregon State University, 2900 SW Campus Way, Corvallis, 97331, Oregon, USA.

*Author to whom correspondence should be addressed:

Linus Pauling Institute and Department of Pharmaceutical Sciences

College of Pharmacy, Oregon State University

2900 SW Campus Way, Corvallis, OR 97331

Tel: 541-737-5078

FAX: 541-737-5077

Email: richard.vanbreemen@oregonstate.edu

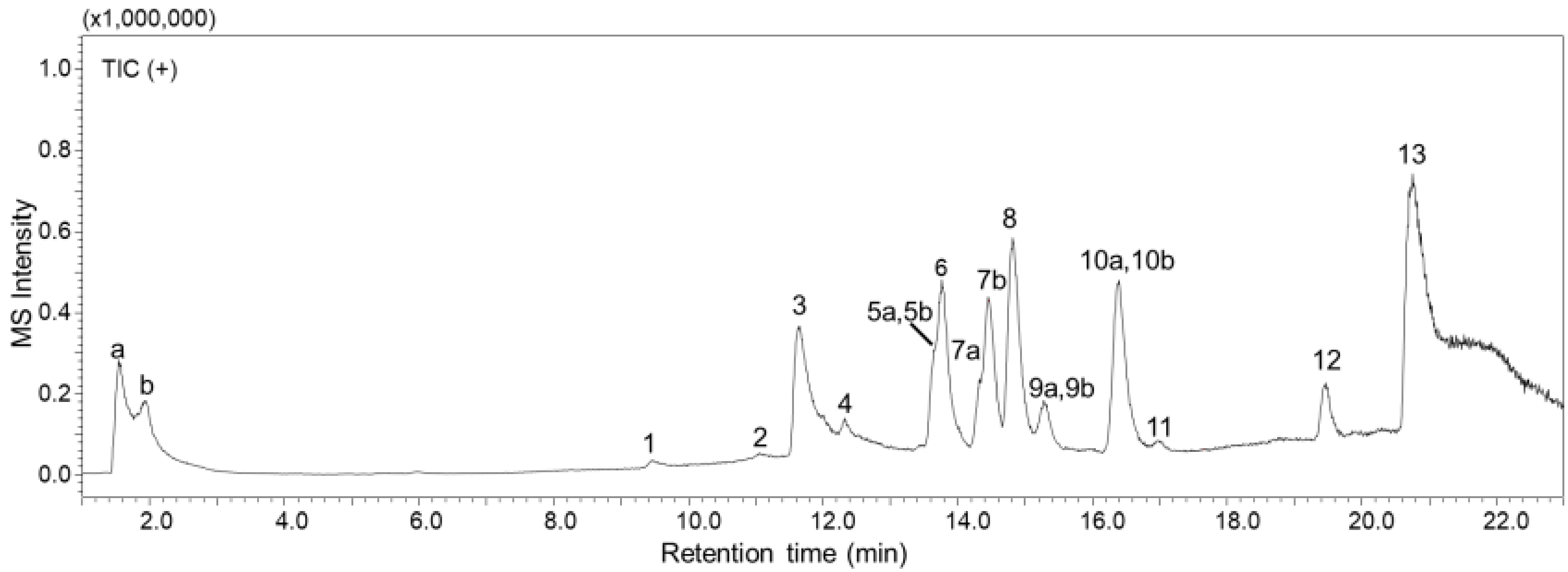

Figure S1. Total ion chromatogram of kava extract (10 µg/mL) analyzed on a Shimadzu 9030 UHPLC-Q-ToF mass spectrometer (resolving power 30,000) with positive ion electrospray. A 25-min gradient from 5% to 95% methanol containing formic acid (0.1%) in water (also containing 0.1% formic acid) was used for the UHPLC separation with a Waters Cortecs C_{18} (2.1 × 150 mm, 1.7 µm) column.

.

Table 1. Proposed and confirmed components corresponding to the peaks in the UHPLC-HRMS chromatogram in **Figure S1** of Kava (*Piper methysticum*) extract.

Peak number	Retention time (min)	*m/z* $[M+H]^+$	ΔM (ppm error)	Proposed ID Molecular Formula (CAS) Confidence[3]	Chemical Structure	Comments
1	9.53	259.0882	7.7	Daidzein-d4<br>$C_{15}H_6D_4O_4$<br>486-66-8<br>Reference standard	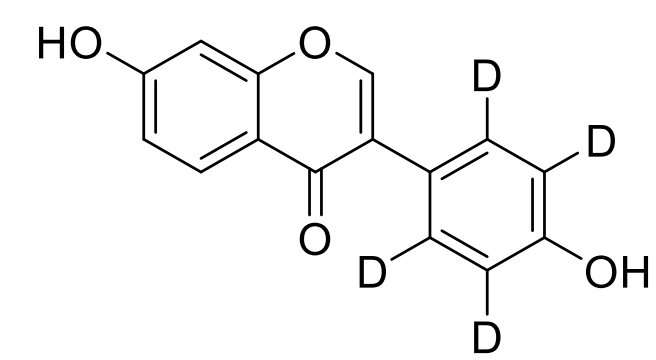	HRMS supports molecular formula. MS/MS supports structure via reference standard. Retention time matches that of reference standard. Daidzein-d4 was included as an injection standard.
2	10.97	279.1200	9.4	11-Hydroxy-12-methoxydihydrokavain<br>$C_{15}H_{18}O_5$<br>-<br>Tentative	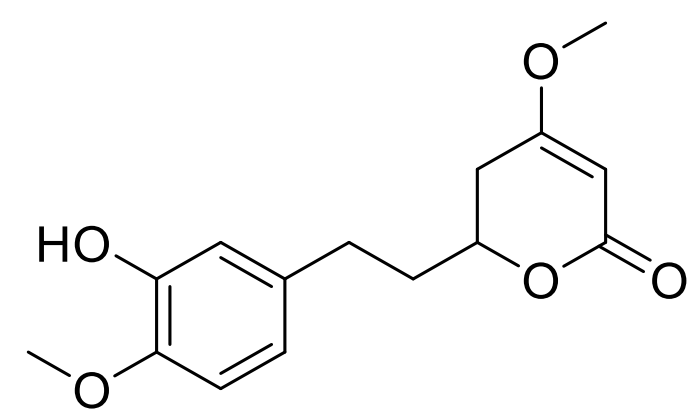	HRMS supports molecular formula. MS/MS supports structure where the major fragment ions are also observed in other kavalactones. This structure is reported in the literature [1].
3	11.53	201.2392	-	Unknown	-	The observed peak is a background noise that was also observed in the solvent. When the chromatogram was aligned with the XCMS online, the fold difference between the peak in the extract and the blank was insignificant
4	12.35	293.1364	6.6	11,12-Dimethoxydihydrokavain<br>$C_{16}H_{20}O_5$<br>-<br>Partial	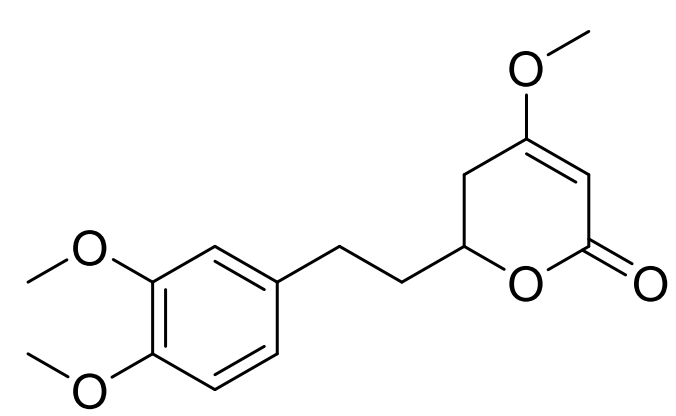	The HRMS support molecular formulae. The structure is partially supported by the MS/MS via manual interpretation. It might be another kavalactone that is not reported in the literature.

Peak number	Retention time (min)	*m/z* [M+H]+	ΔM (ppm error)	Proposed ID Molecular Formula (CAS) Confidence[3]	Chemical Structure	Comments
5a	13.59	289.1050	7.1	10-Methoxyyangonin<br>$C_{16}H_{16}O_5$<br>77900-32-4<br>Partial	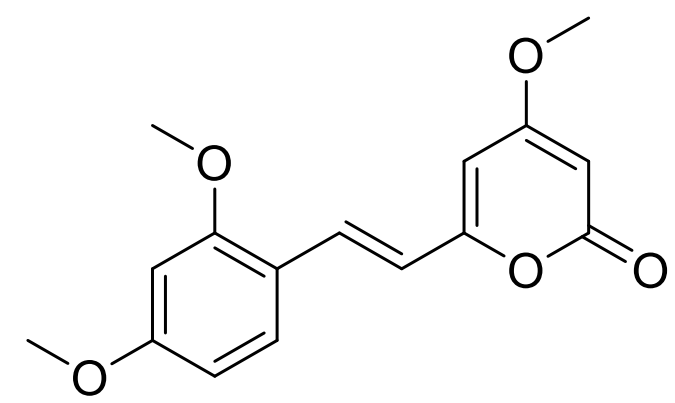	The HRMS support molecular formulae. The structure is partially supported by the MS/MS via manual interpretation. It might be another kavalactone that is not reported in the literature.
**5b**	13.67	275.0895	6.9	Methysticin<br>$C_{15}H_{14}O_5$<br>495-85-2<br>Reference standard	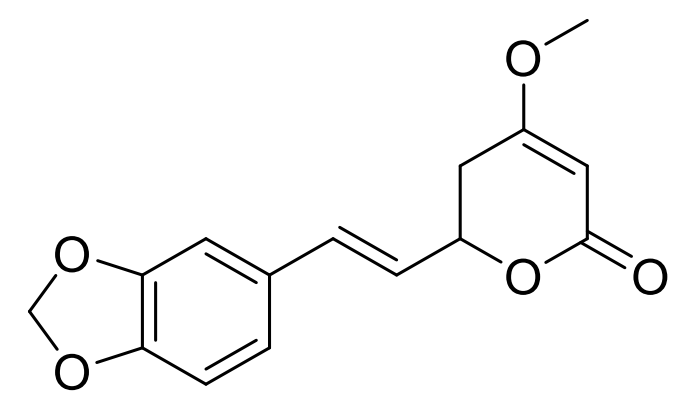	HRMS supports molecular formula. MS/MS supports structure via reference standard.<br>Retention time matches that of reference standard
**6**	13.79	277.1052	6.8	7,8-Dihydromethysticin<br>$C_{15}H_{16}O_5$<br>19902-91-1<br>Reference standard	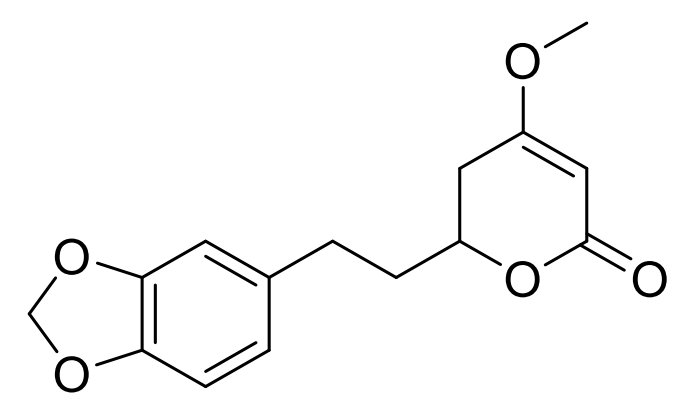	HRMS supports molecular formula. MS/MS supports structure via reference standard.<br>Retention time matches that of reference standard
7a	14.32	263.1260	6.7	5,6,7,8-Tetrahydroyangonin<br>$C_{15}H_{18}O_4$<br>-<br>Matched	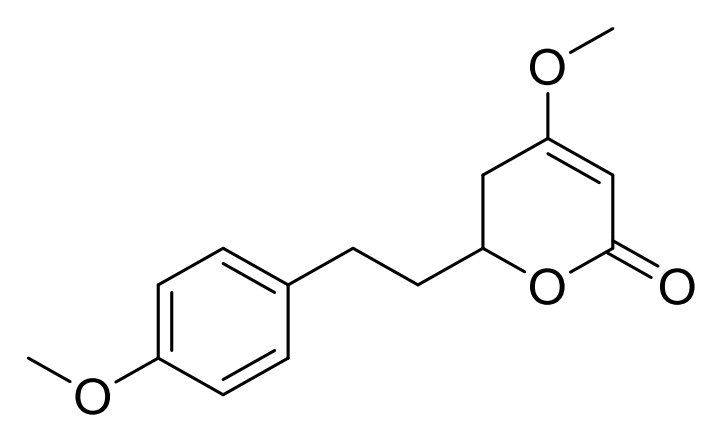	HRMS supports molecular formula. MS/MS supports structure via manual interpretation where the major fragment ions are also observed in other kavalactones. The MS/MS matches that reported in the literature. [2].

**Peak number**	**Retention time (min)**	***m/z* [M+H]$^+$**	**ΔM (ppm error)**	**Proposed ID Molecular Formula (CAS) Confidence[3]**	**Chemical Structure**	**Comments**
**7b**	14.48	231.1001	6.5	Kavain<br>$C_{14}H_{14}O_3$<br>500-64-1<br>Reference standard	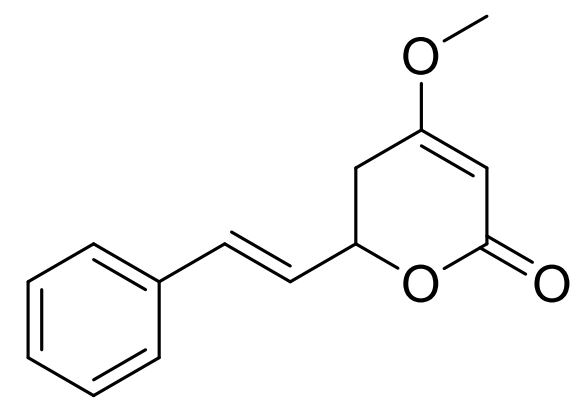	HRMS supports molecular formula.<br>MS/MS supports structure via reference standard.<br>Retention time matches that of reference standard
**8**	14.86	233.1159	5.6	7,8-Dihydrokavain<br>$C_{14}H_{16}O_3$<br>587-63-3<br>Reference standard	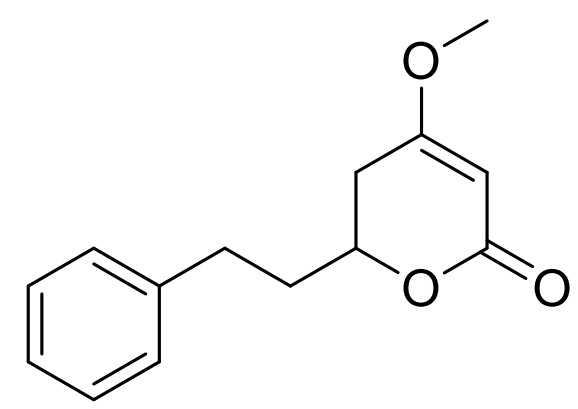	HRMS supports molecular formula.<br>MS/MS supports structure via reference standard.<br>Retention time matches that of reference standard
**9a**	15.33	259.0947	6.9	Yangonin<br>$C_{15}H_{14}O_4$<br>500-62-9<br>Reference standard	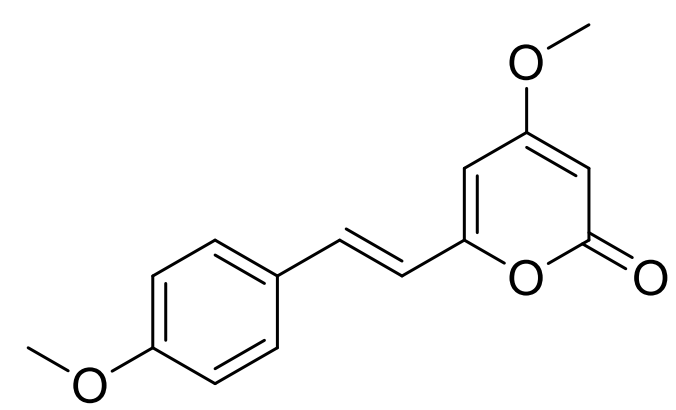	HRMS supports molecular formula.<br>MS/MS supports structure via reference standard.<br>Retention time matches that of reference standard
9b	15.33	262.1135	6.8	Yangonin-*d3*<br>$C_{15}H_{11}D_3O_4$<br>500-62-9<br>Reference standard	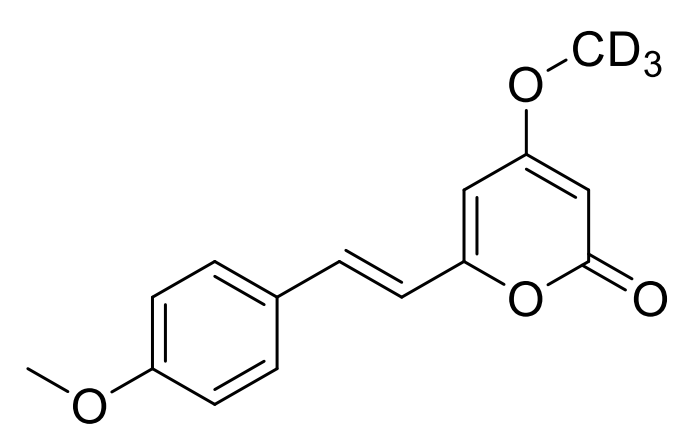	HRMS supports molecular formula.<br>MS/MS supports structure via reference standard.<br>Retention time matches that of reference standard. This compound was used as an internal standard.

Peak number	Retention time (min)	*m/z* $[M+H]^+$	ΔM (ppm error)	Proposed ID Molecular Formula (CAS) Confidence[3]	Chemical Structure	Comments
**10a**	16.35	229.0844	6.5	Desmethoxyyangonin<br>$C_{14}H_{12}O_3$<br>15345-89-8<br>Reference standard	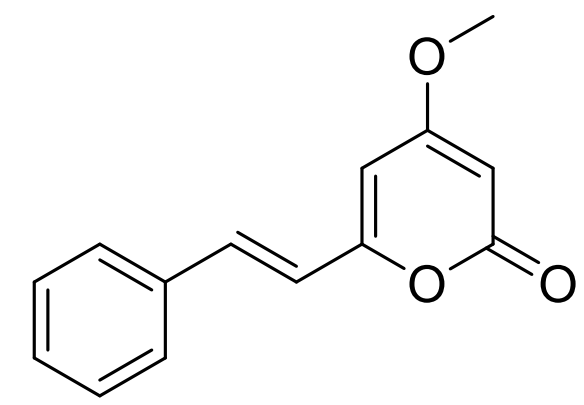	HRMS supports molecular formula. MS/MS supports structure via reference standard. Retention time matches that of reference standard
**10b**	16.61	315.1205	6.9	Flavokavain A<br>$C_{18}H_{18}O_5$<br>3420-72-2<br>Reference standard	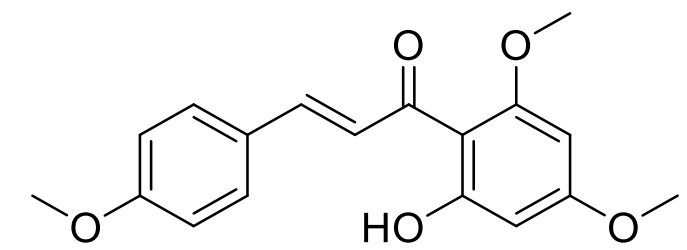	HRMS supports molecular formula. MS/MS supports structure via reference standard. Retention time matches that of reference standard
**11**	17.02	285.1101	7.0	Flavokavain B<br>$C_{17}H_{16}O_4$<br>1775-97-9<br>Reference standard	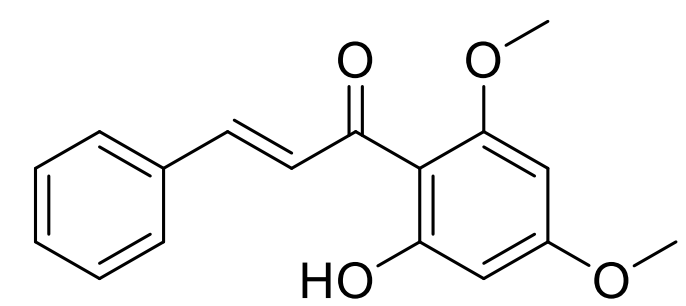	HRMS supports molecular formula. MS/MS supports structure via reference standard. Retention time matches that of reference standard
12	19.49	393.2832		Unknown	-	The observed peak is a background noise that was also observed in the solvent. When the chromatogram was aligned with the XCMS online, the fold difference between the peak in the extract and the blank was insignificant

Peak number	Retention time (min)	*m/z* [M+H]$^+$	ΔM (ppm error)	Proposed ID Molecular Formula (CAS) Confidence[3]	Chemical Structure	Comments
13	20.76	270.3142		Unknown	-	The observed peak is a background noise that was also observed in the solvent. When the chromatogram was aligned with the XCMS online, the fold difference between the peak in the extract and the blank was insignificant

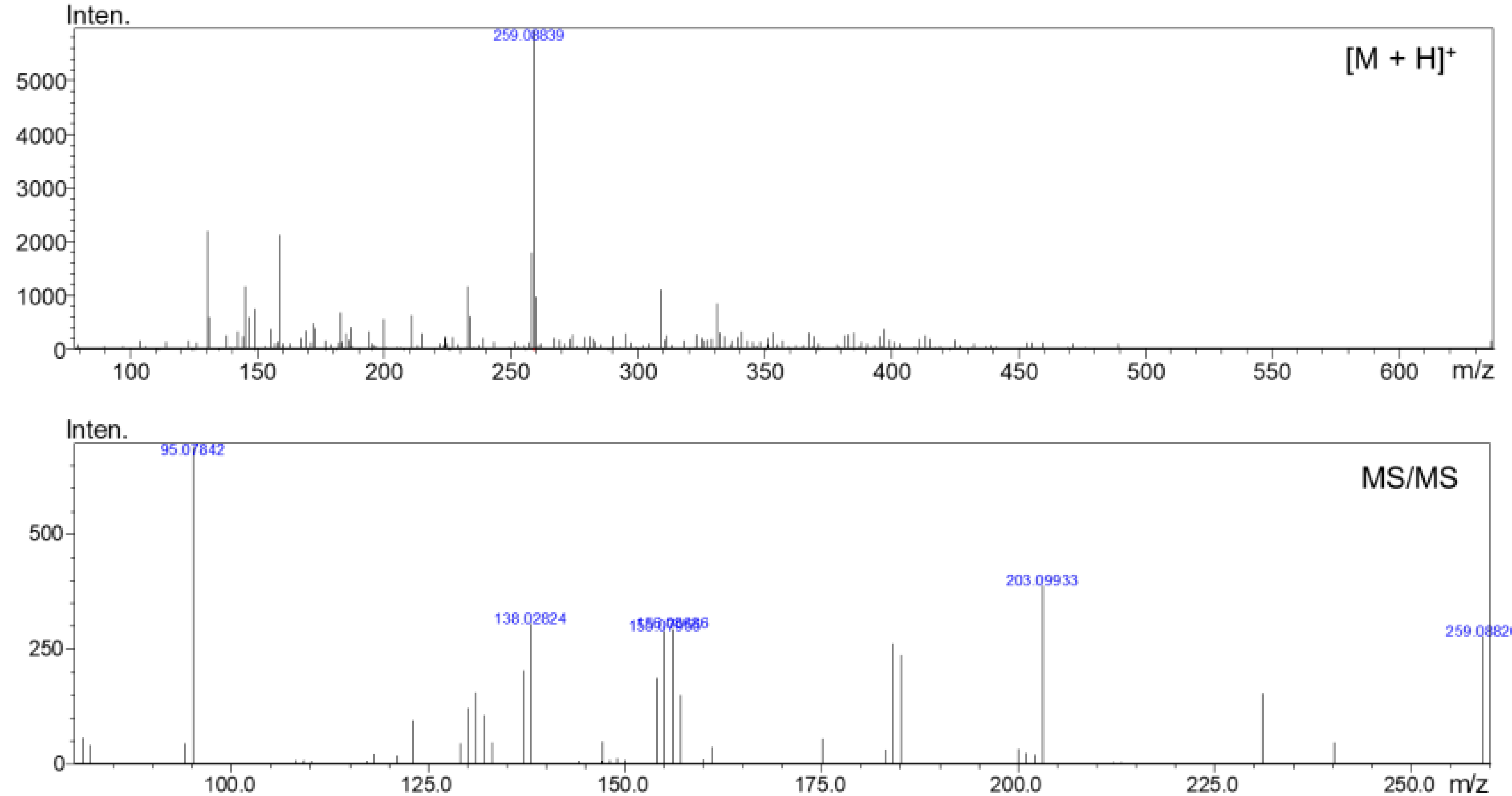

Figure S2. Positive ion electrospray HRMS (top panel) and MS/MS of peak 1 of daidzein-*d4* (internal standard) with *m*/*z* 259.0882 eluting at 9.53 min in Figure S1

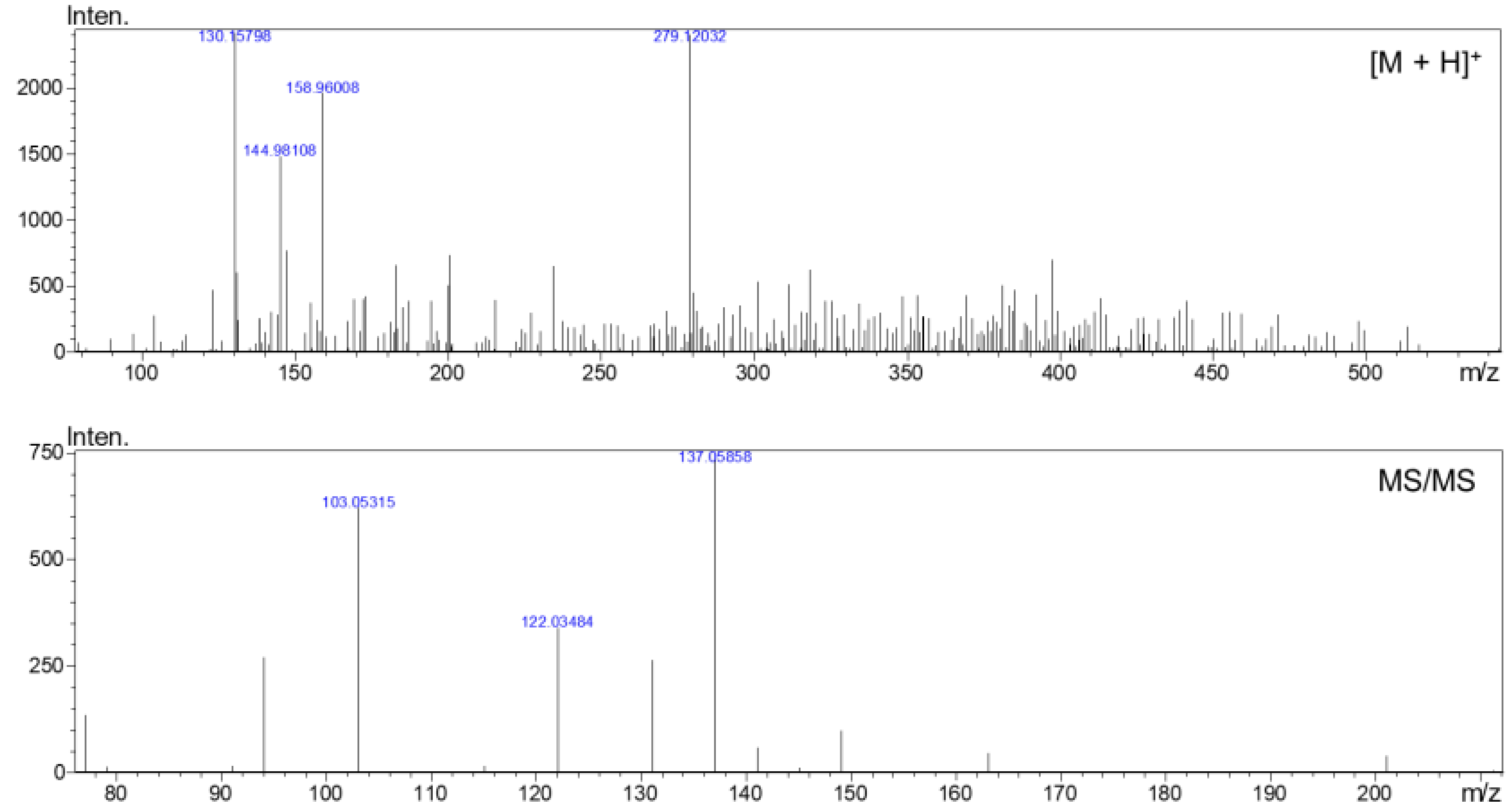

Figure S3. Positive ion electrospray HRMS (top panel) and MS/MS of peak 2 with *m*/*z* 279.1200 eluting at 10.97 min

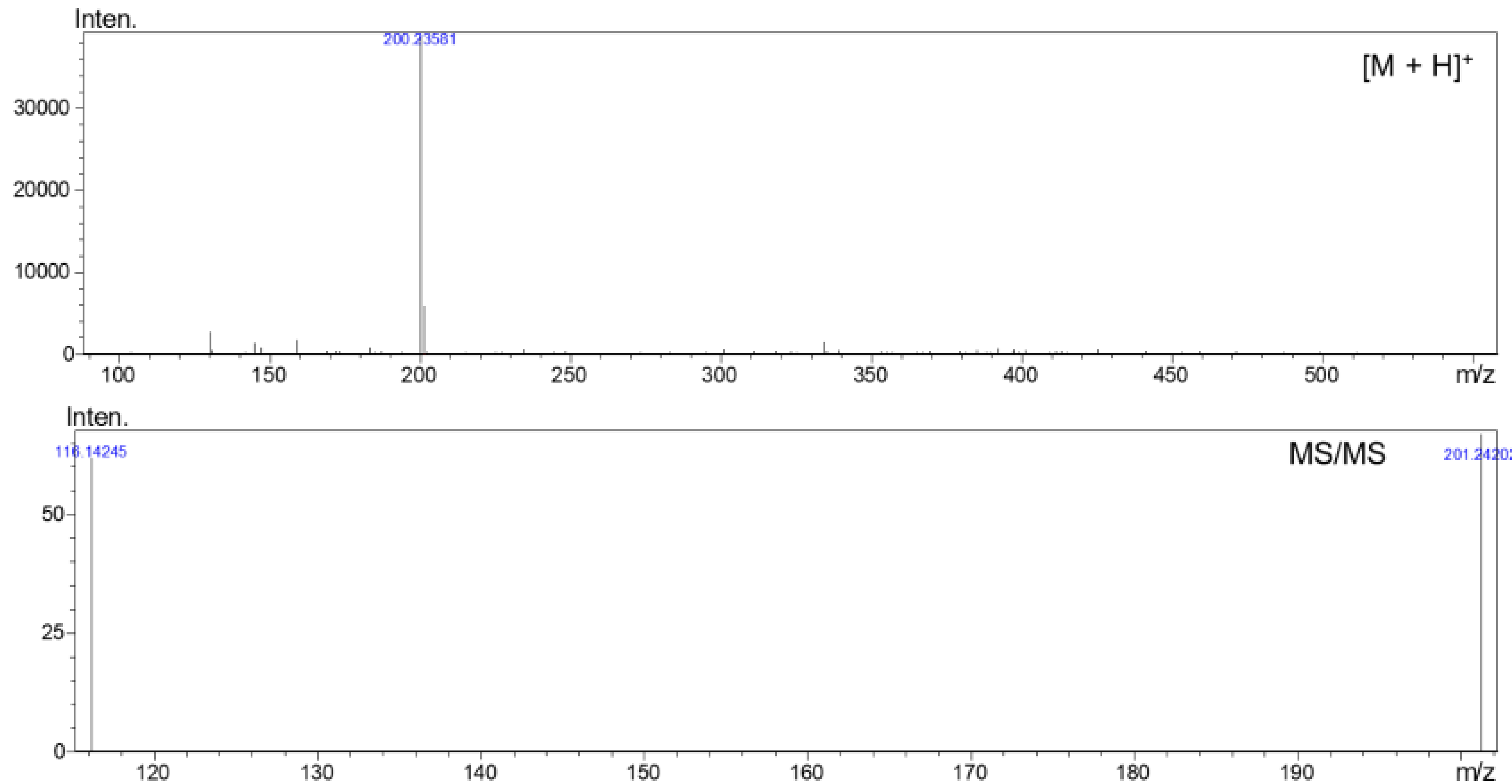

Figure S4. Positive ion electrospray HRMS (top panel) and MS/MS of peak 3 with *m*/*z* 201.2392 eluting at 11.53 min

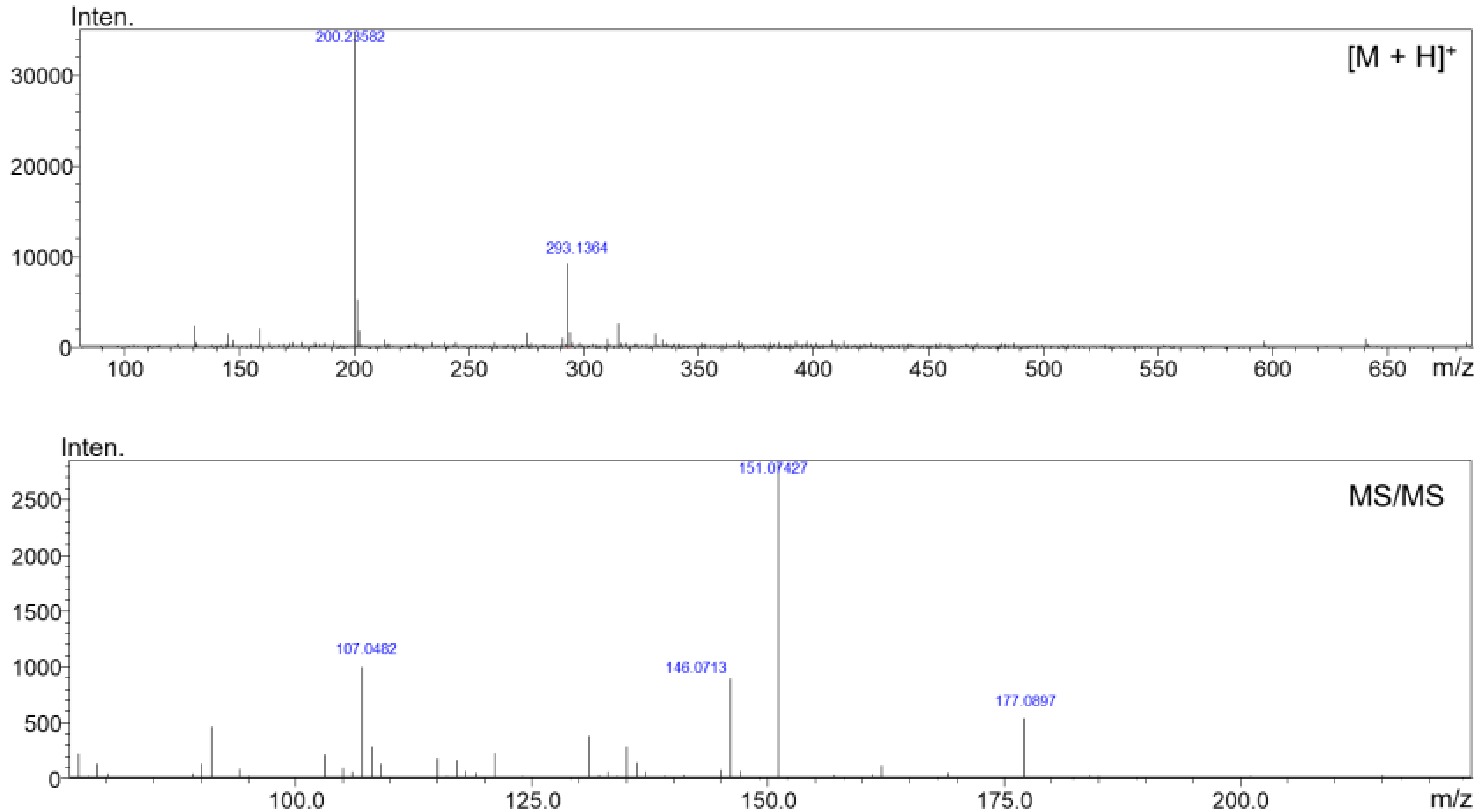

Figure S5. Positive ion electrospray HRMS (top panel) and MS/MS of peak 4 with *m*/*z* 293.1364 eluting at 12.35 min

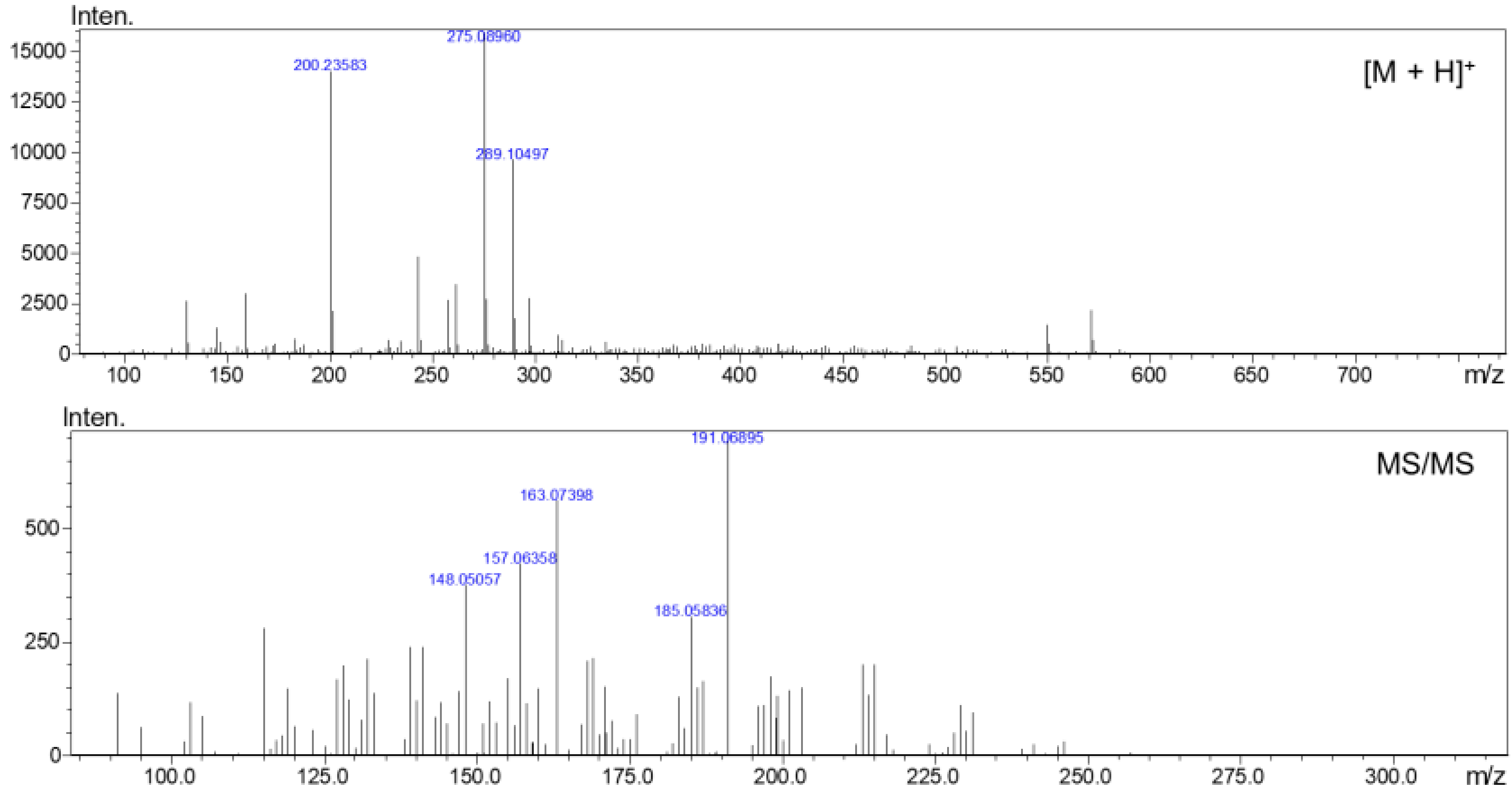

Figure S6. Positive ion electrospray HRMS (top panel) and MS/MS of peak 5a with *m/z* 289.1050 eluting at 13.59 min

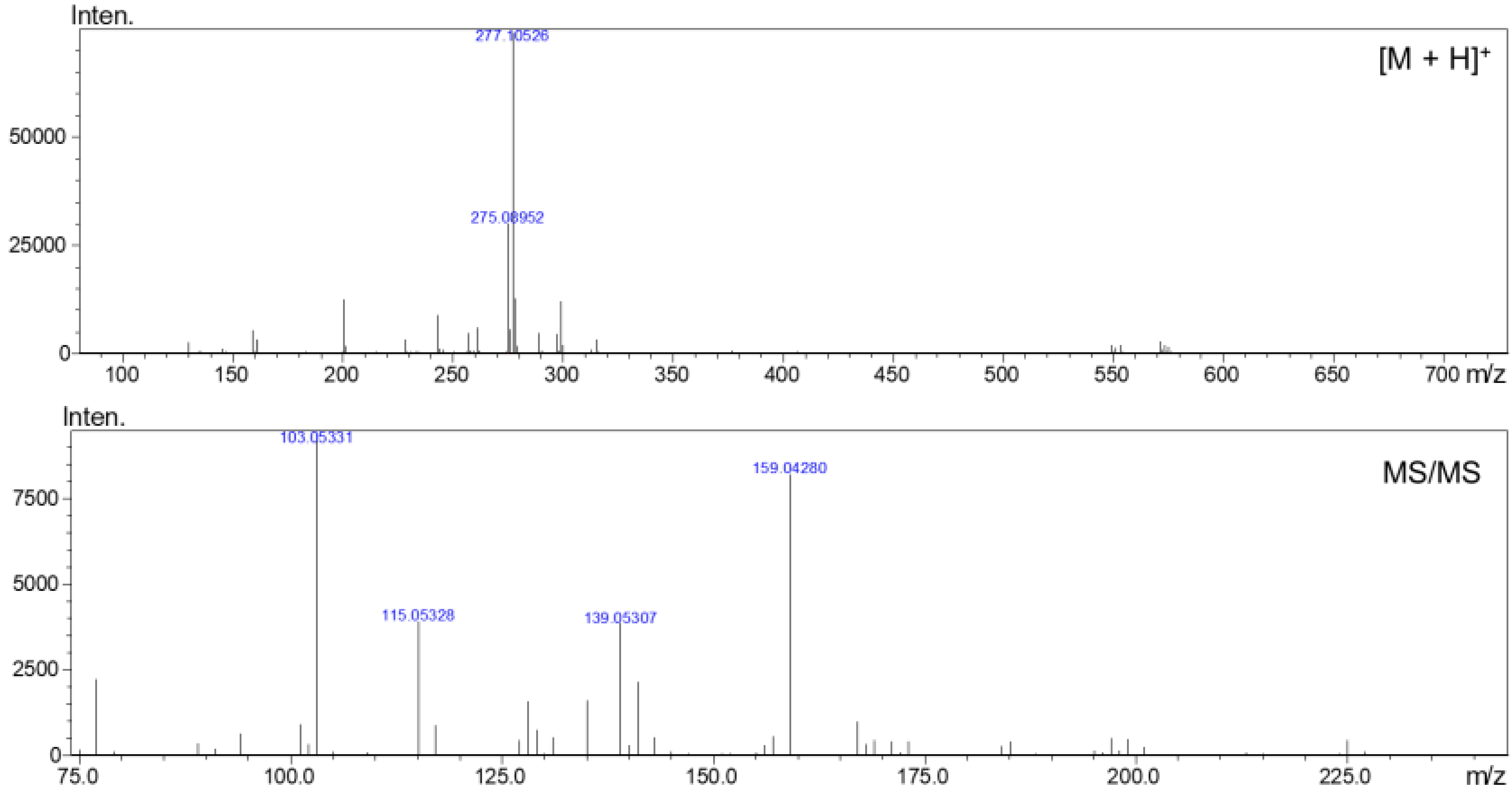

Figure S7. Positive ion electrospray HRMS (top panel) and MS/MS of peak 5b (methysiticin) with *m*/*z* 275.0895 eluting at 13.67 min

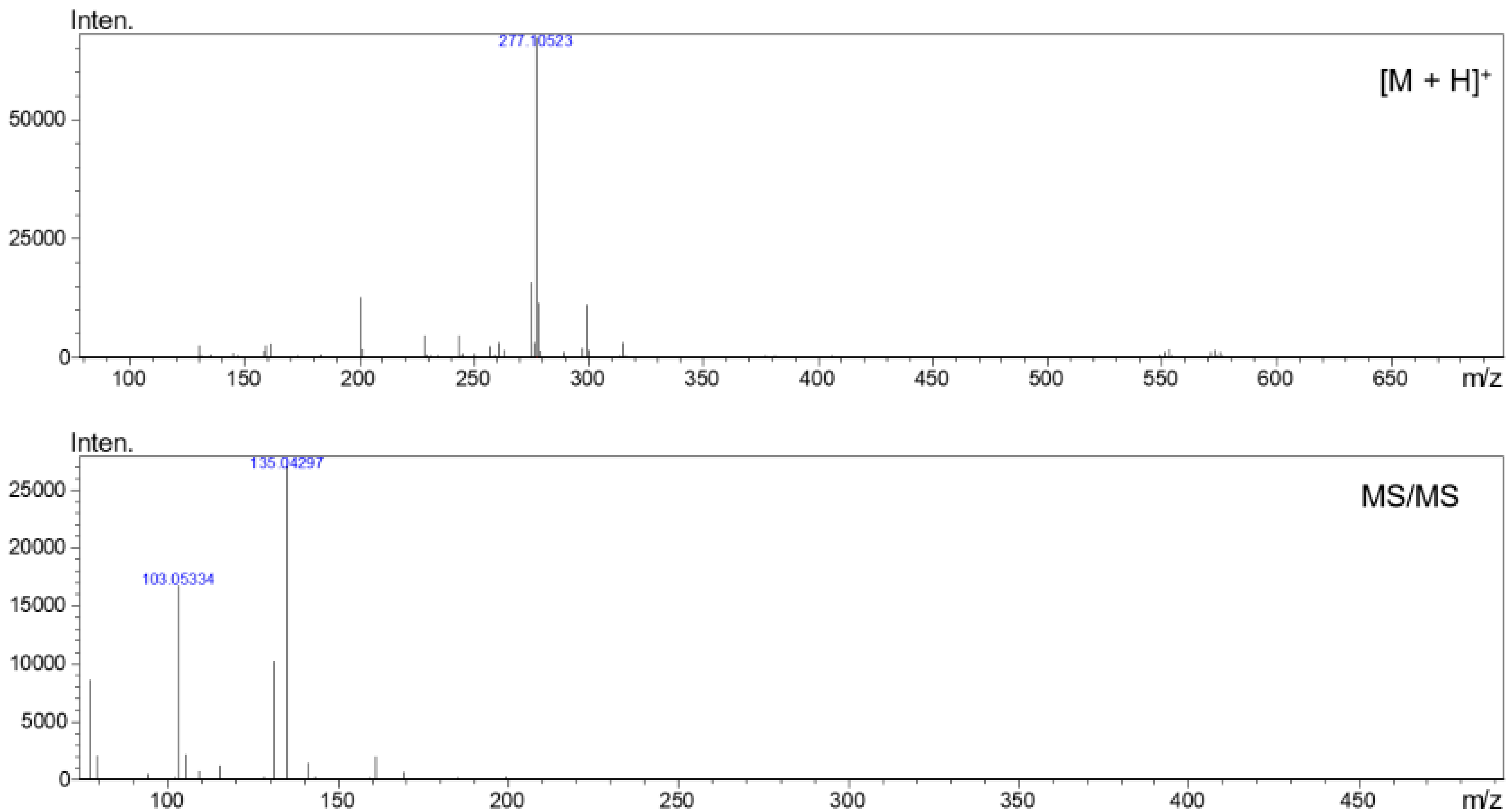

Figure S8. Positive ion electrospray HRMS (top panel) and MS/MS of peak 6 (7,8-dihydromethysticin) with *m/z* 277.10523 eluting at 13.79 min

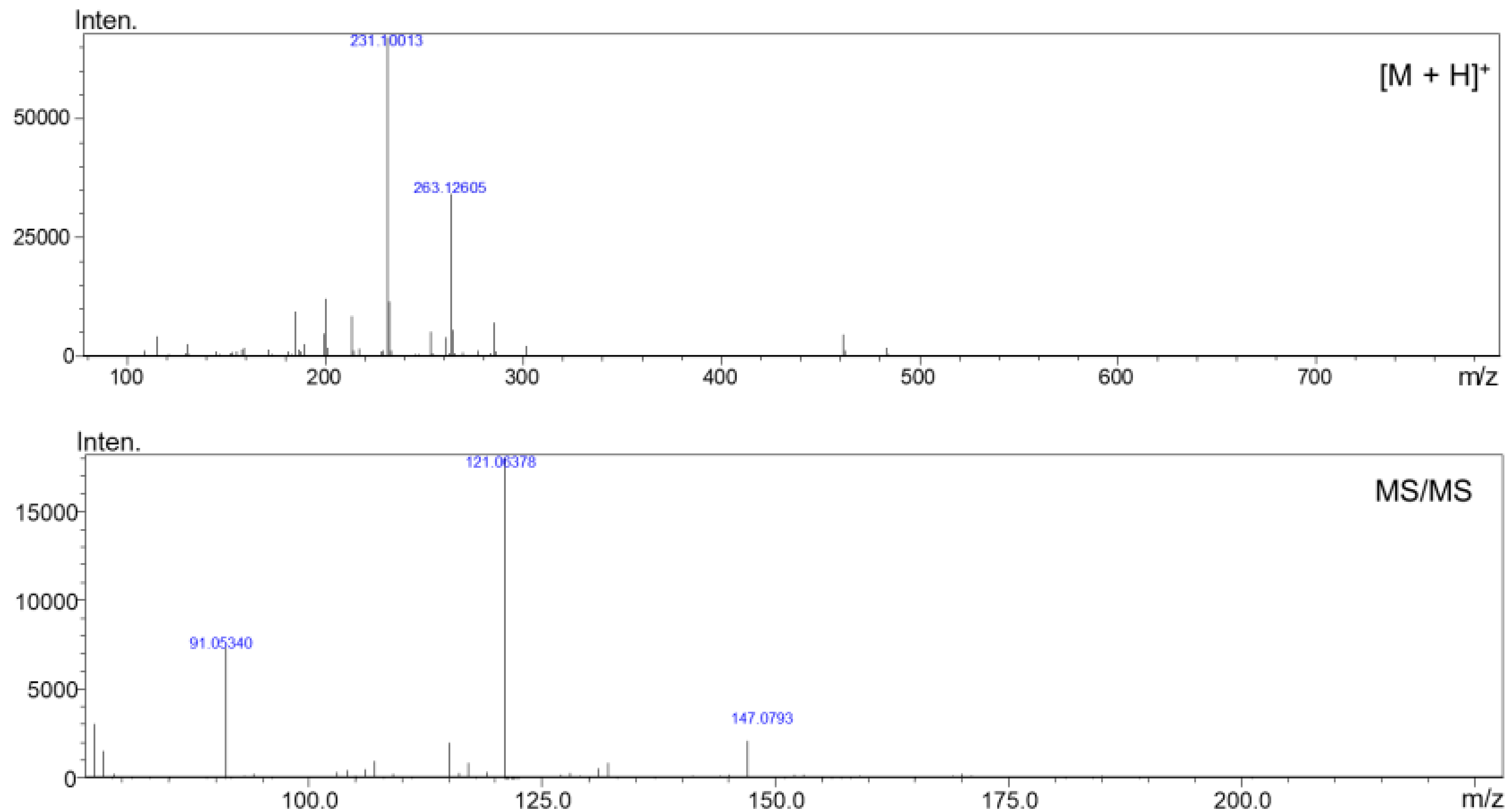

Figure S9. Positive ion electrospray HRMS (top panel) and MS/MS of peak 7a with *m/z* 263.1260 eluting at 14.32 min

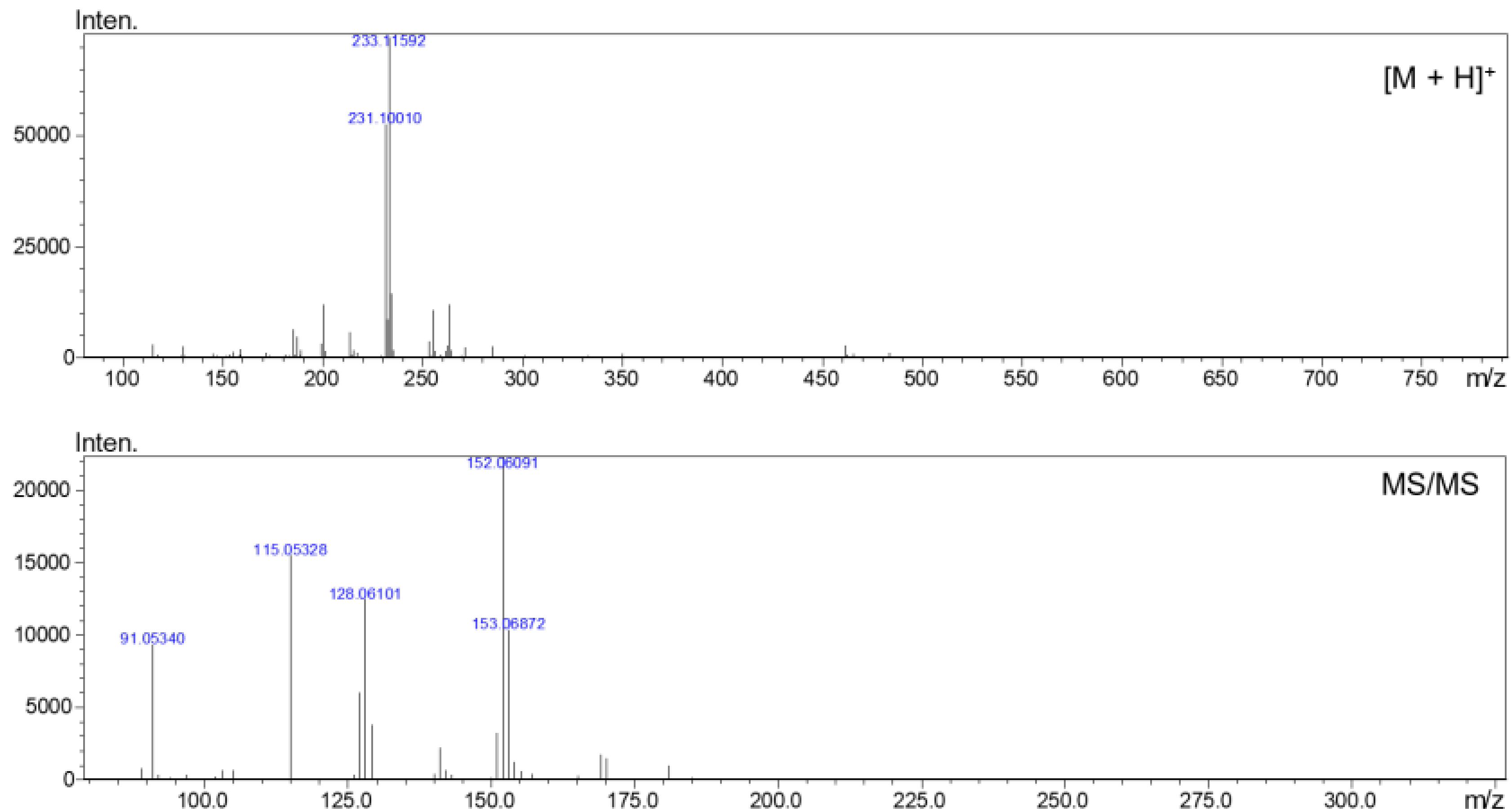

Figure S10. Positive ion electrospray HRMS (top panel) and MS/MS of peak 7b (kavain) with *m*/*z* 231.1001 eluting at 14.48 min

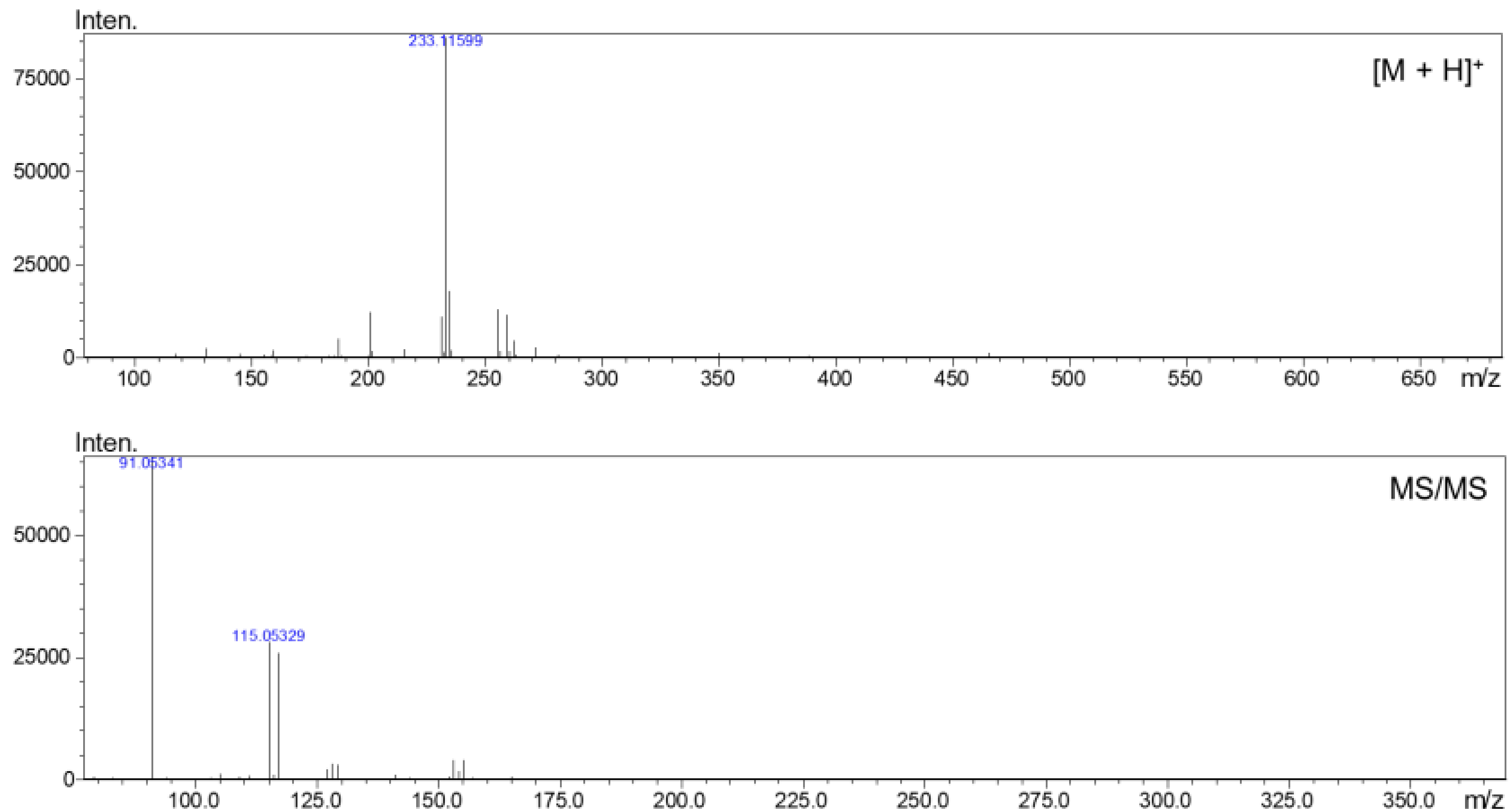

Figure S11. Positive ion electrospray HRMS (top panel) and MS/MS of peak 8 (7,8-dihydrokavain) with *m/z* 233.1159 eluting at 14.86 min

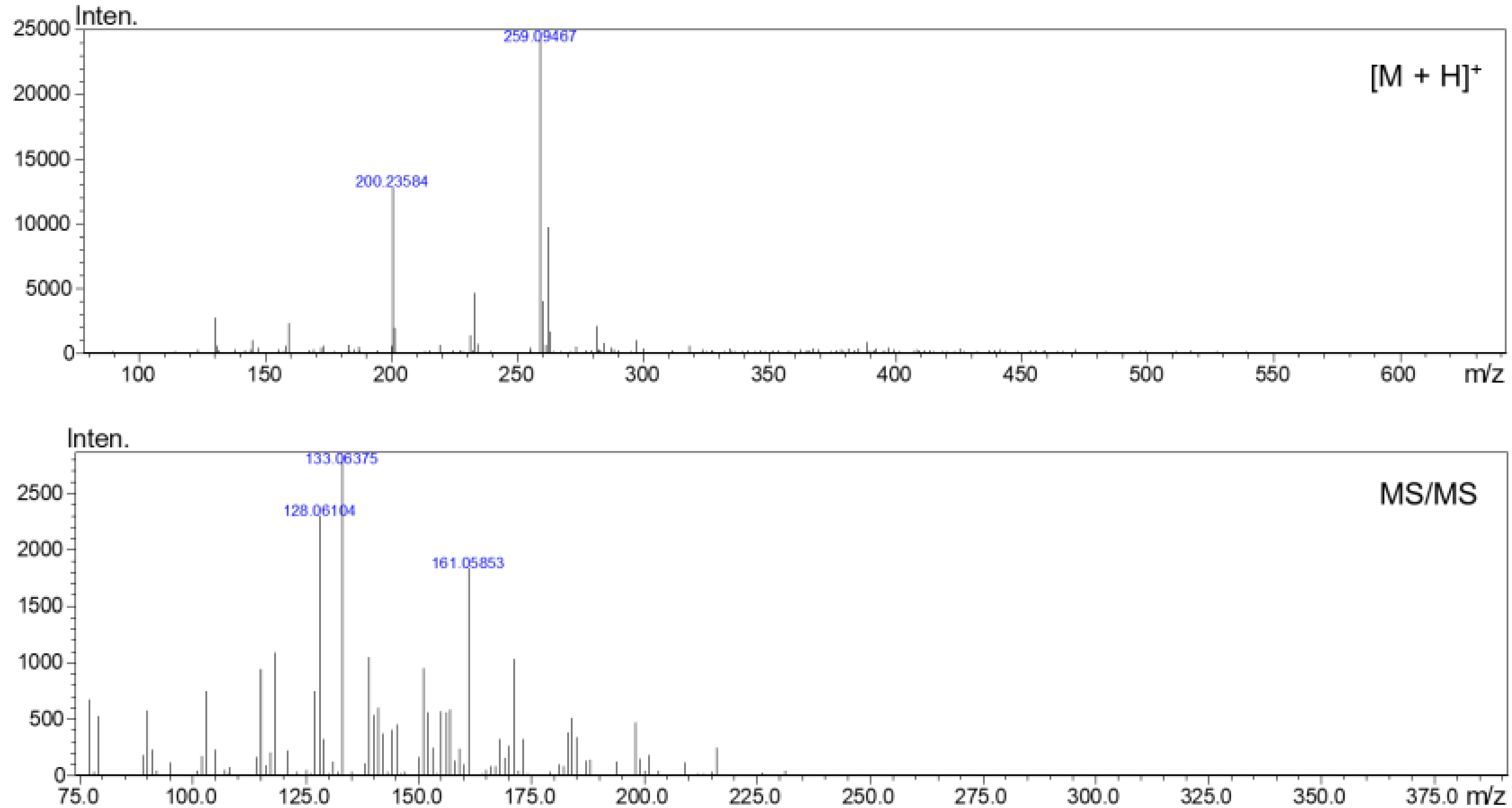

Figure S12. Positive ion electrospray HRMS (top panel) and MS/MS of peak 9a with *m*/*z* 259.0947 eluting at 15.33 min

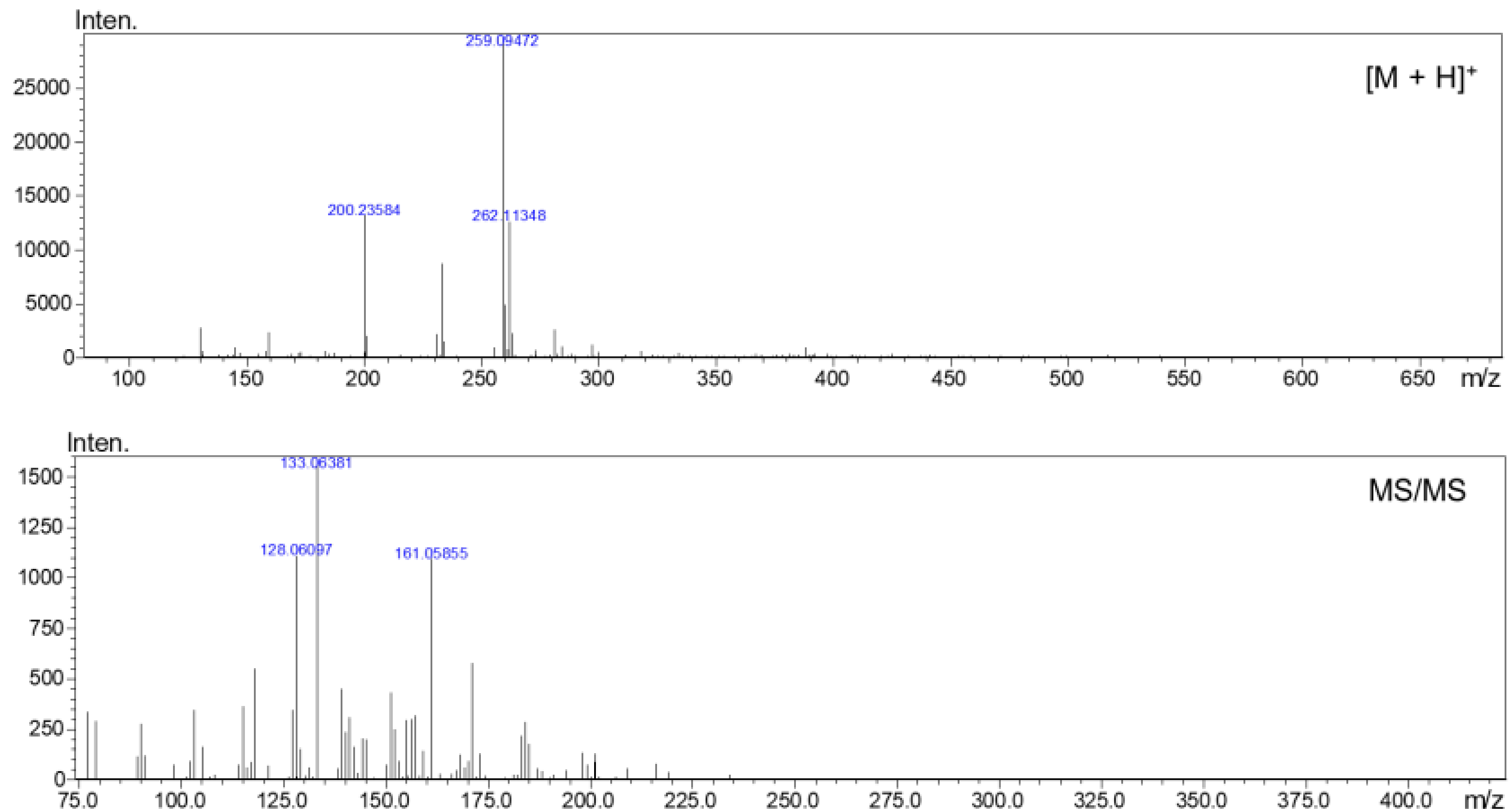

Figure S13. Positive ion electrospray HRMS (top panel) and MS/MS of peak 9b (yangonin-*d3*) with *m*/*z* 262.1135 eluting at 15.33 min

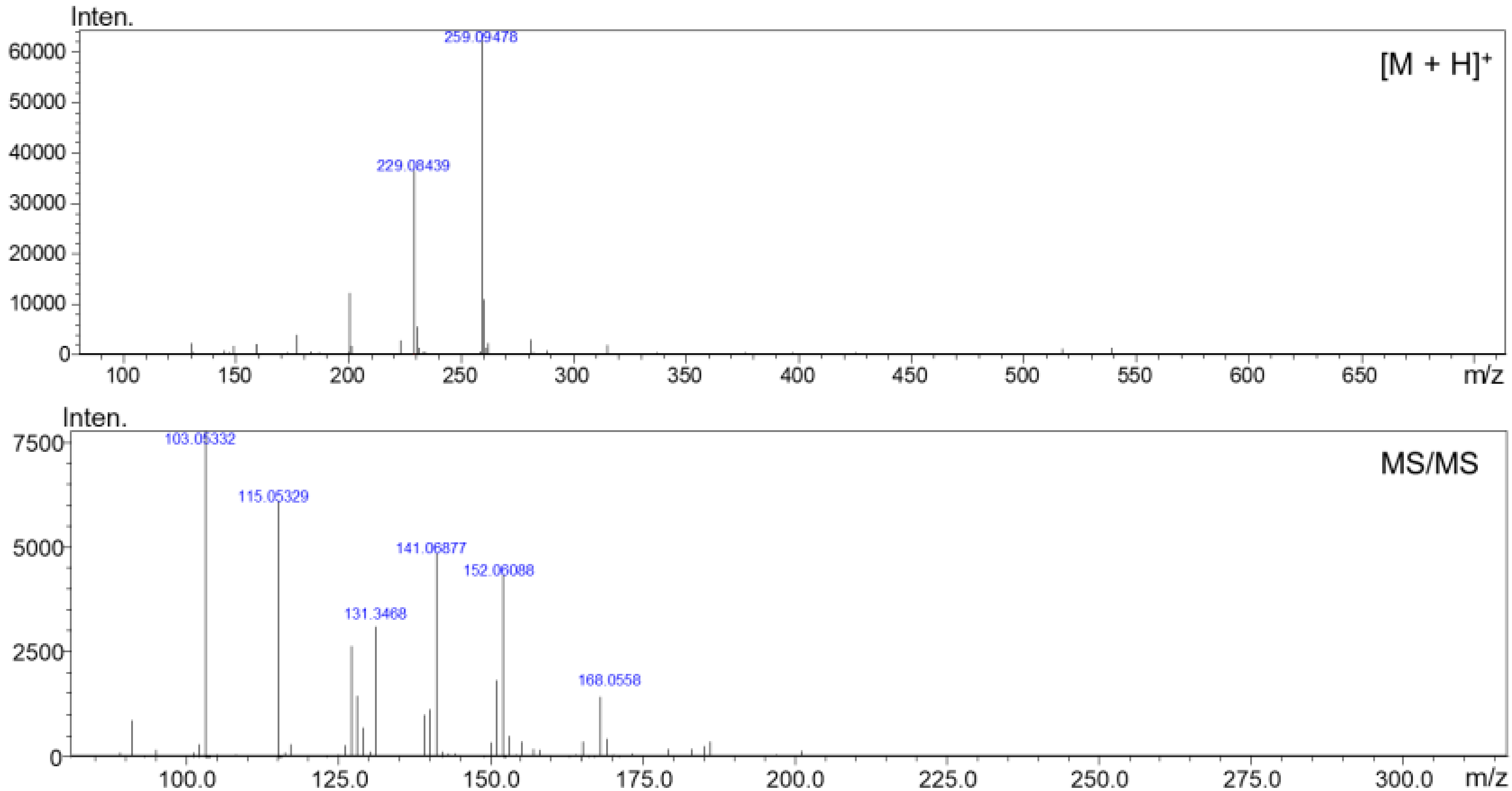

Figure S14. Positive ion electrospray HRMS (top panel) and MS/MS of peak 10a (desmethoxyyangonin) with *m/z* 229.0844 eluting at 16.35 min

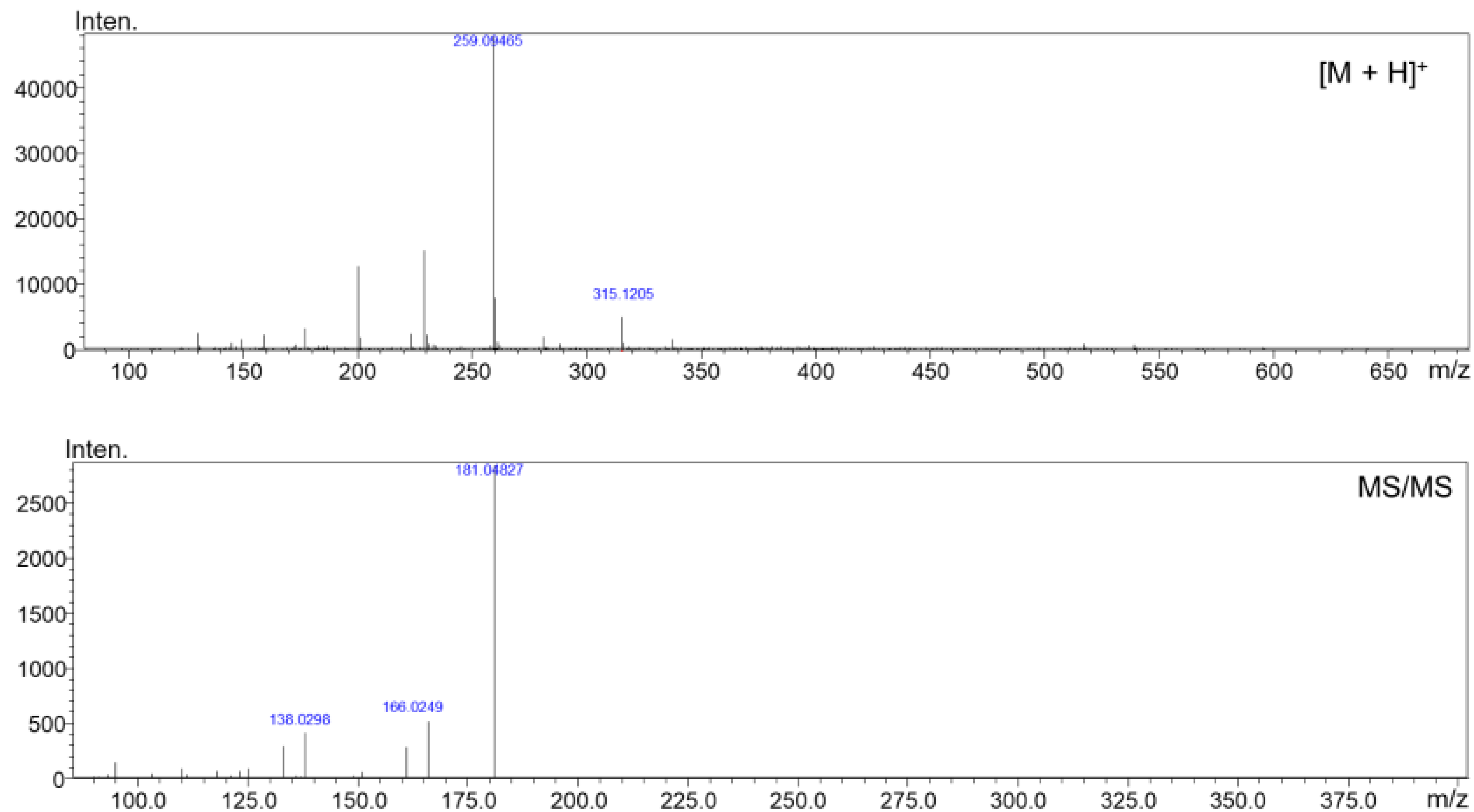

Figure S15. Positive ion electrospray HRMS (top panel) and MS/MS of peak 10b (flavokavain A) with *m/z* 315.1205 eluting at 16.61 min

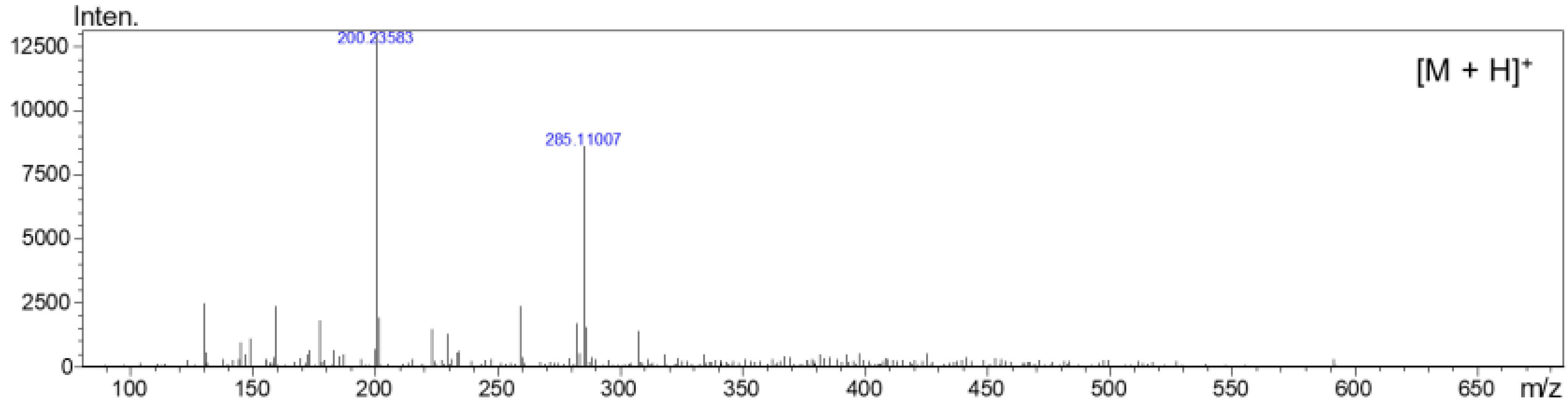

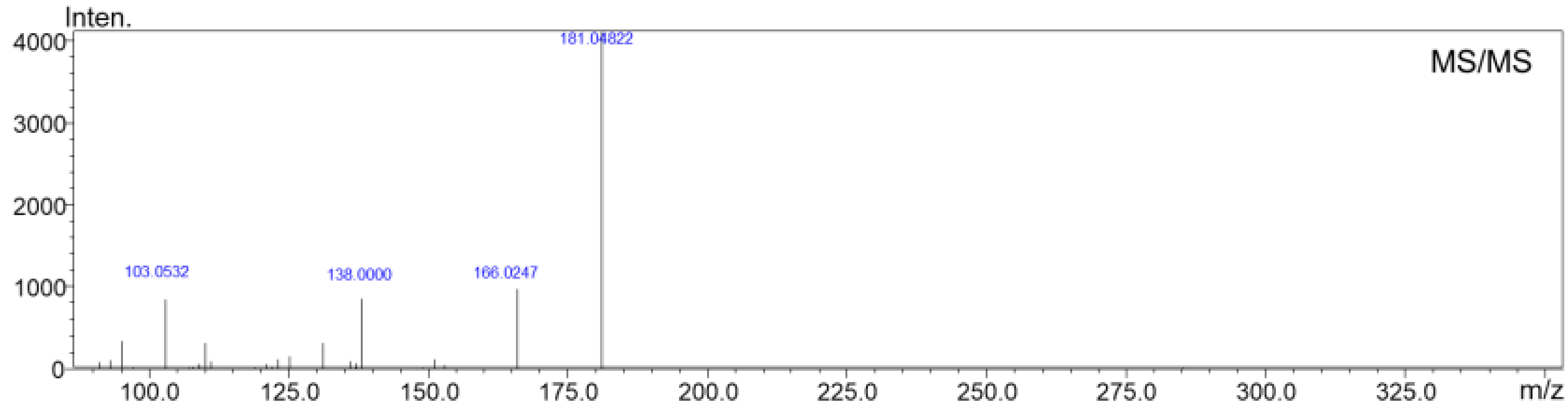

Figure S16. Positive ion electrospray HRMS (top panel) and MS/MS of peak 11 (flavokavain B) with *m*/*z* 285.1101 eluting at 17.02 min

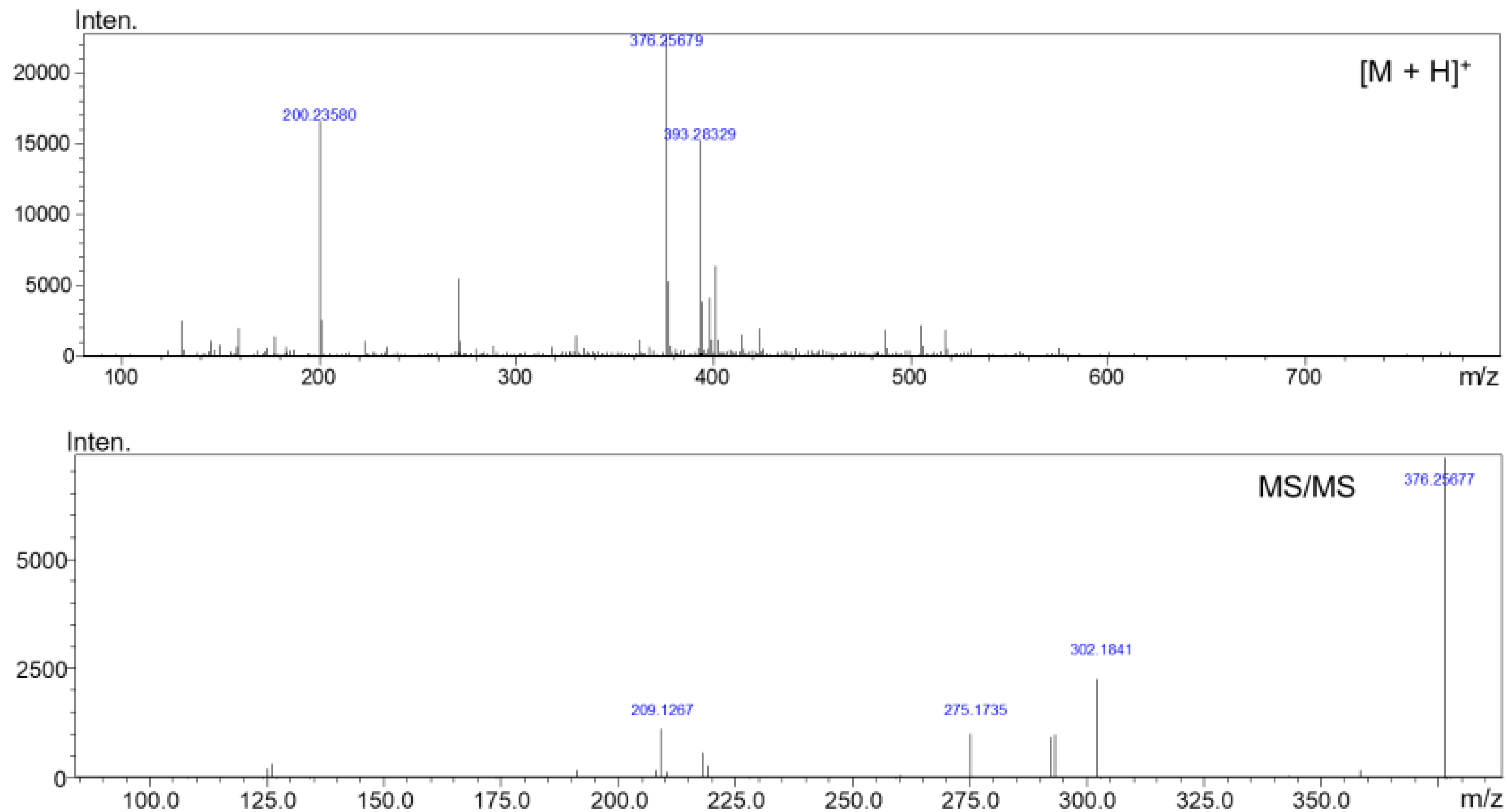

Figure S17. Positive ion electrospray HRMS (top panel) and MS/MS of peak 12 with *m*/*z* 393.2832 eluting at 19.49 min

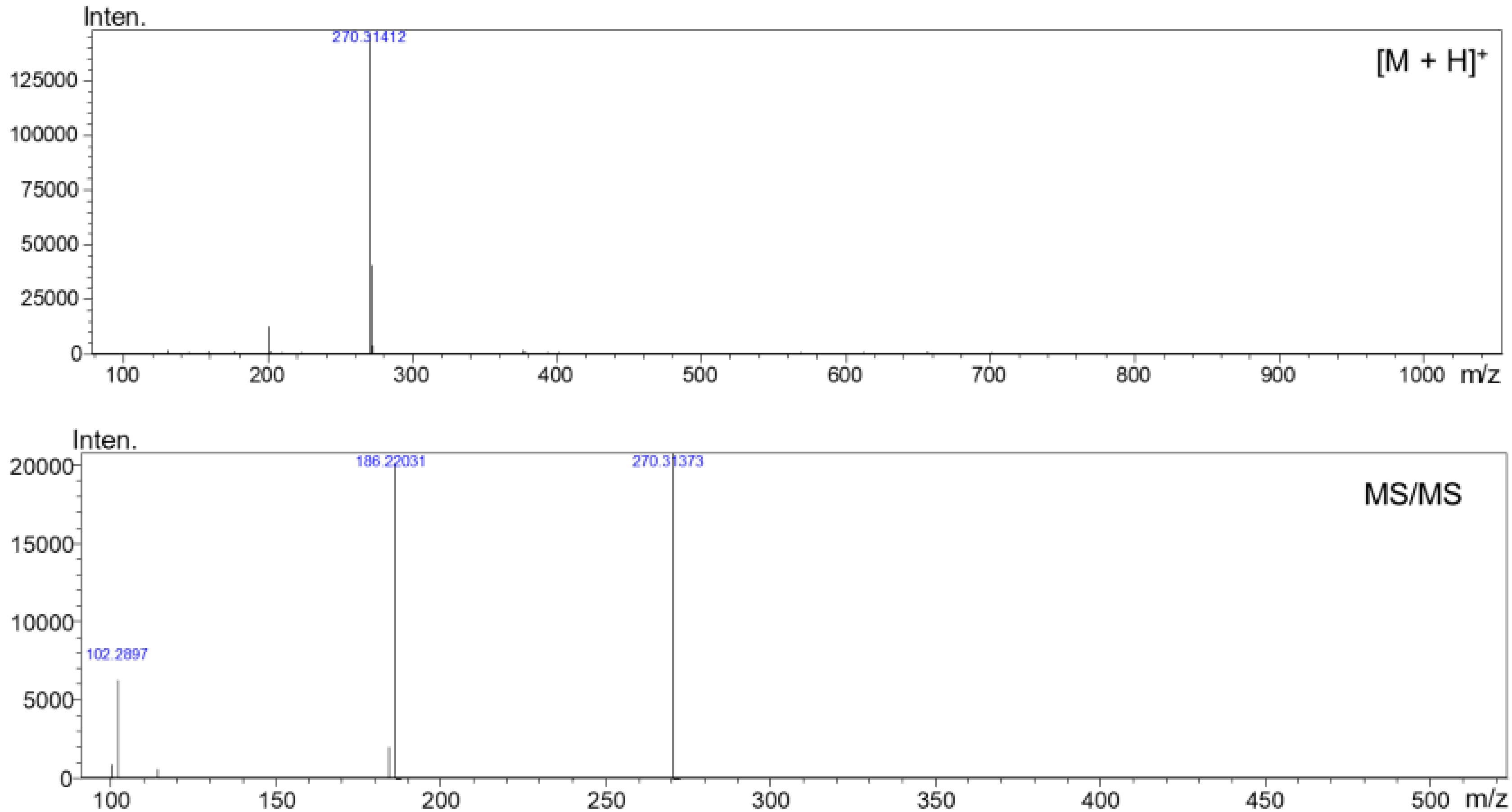

Figure S18. Positive ion electrospray HRMS (top panel) and MS/MS of peak 13 with *m/z* 270.3142 eluting at 20.76 min

References

[1] X.-G. He, L.-Z. Lin, L.-Z. Lian. Electrospray high performance liquid chromatography-mass spectrometry in phytochemical analysis of kava (*Piper methysticum*) extract. *Planta Medica*. 63 (1997) 70.

[2] Tang Y, Fields CA. UHPLC-UV method development and validation for determining kavalactones and flavokavains in *Piper methysticum* (kava). *Molecules*. 24 (2019) 1245.
